# Mild Hypogammaglobulinemia Can Be a Serious Condition

**DOI:** 10.3389/fimmu.2018.02384

**Published:** 2018-10-15

**Authors:** Lisanne M. A. Janssen, Paul Bassett, Thomas Macken, Jolanda van Esch, Hans Pruijt, Arnoud Knoops, Markus Sköld, Antony Parker, Jolanda de Vries, Esther de Vries

**Affiliations:** ^1^Department of Pediatrics, Amalia Children's Hospital, Nijmegen, Netherlands; ^2^Stats Consultancy Ltd. Amersham, United Kingdom; ^3^Department of Pulmonology, Jeroen Bosch Hospital, ‘s-Hertogenbosch, Netherlands; ^4^Department of Pediatrics, Jeroen Bosch Hospital, ‘s-Hertogenbosch, Netherlands; ^5^Department of Internal Medicine, Jeroen Bosch Hospital, ‘s-Hertogenbosch, Netherlands; ^6^Department of Radiology, Jeroen Bosch Hospital, ‘s-Hertogenbosch, Netherlands; ^7^The Binding Site Group Limited, Birmingham, United Kingdom; ^8^Department of Medical and Clinical Psychology, Tilburg University and Elisabeth TweeSteden Hospital, Tilburg, Netherlands; ^9^Department of Tranzo, Tilburg University, Tilburg, Netherlands; ^10^Laboratory for Medical Microbiology and Immunology, Elisabeth Tweesteden Hospital, Tilburg, Netherlands

**Keywords:** primary antibody deficiency, immunodeficiency, unclassified primary antibody deficiency, primary immunodeficiency, hypogammaglobulinemia, common variable immunodeficiency disorders, pneumococcal vaccination response

## Abstract

**Background:** Most patients with primary antibody deficiency (PAD) suffer from less well-described and understood forms of hypogammaglobulinemia (unclassified primary antibody deficiency, unPAD). Because of the moderately decreased immunoglobulin levels compared to CVID, unPAD is generally considered to be clinically mild and not very relevant.

**Objective:** To describe our cohort of—mainly—unPAD patients, and to analyze whether subgroups can be identified.

**Methods:** Data were prospectively collected (February-2012 to June-2016) as part of a standardized, 1-day Care Pathway for suspected primary immunodeficiency. The TNO-AZL Questionnaire for Health-Related Quality of Life (HRQoL) was part of the pre-first-visit intake procedure.

**Results:** Three hundred and twenty patients were referred to the Care Pathway. Data from 23/27 children and 99/113 adults who were diagnosed with PAD and gave informed consent were available for analysis. 89/99 adults had unPAD, the majority (74%) were female and 44% already showed bronchiectasis. HRQoL was significantly decreased in *all* domains, meaning that a lot of unPAD patients had to cope simultaneously with pain, negative feelings and impairments in cognition, home management tasks, sleep, social interaction, and work. The most prominently impaired HRQoL domain was vitality, indicating these patients feel extremely tired and worn out.

**Conclusion:** These results highlight the need for more attention to the potential patient burden of unPADs. A larger cohort is needed to increase our understanding of unPADs and to analyze whether distinct subgroups can be identified. For now, it is important for the clinician to acknowledge the existence of unPAD and be aware of its potential consequences, in order to timely and appropriately manage its effects and complications.

## Introduction

Primary immune deficiencies (PIDs) are rare, inherited defects of the immune system with more than 300 forms described to date (1). Only a small subgroup of patients suffer from a form of PID that leads to significant problems very early in life (2). Most PID patients have PID forms that are less severe and present later in life (1–4), mainly comprising of diseases with predominantly (primary) antibody deficiency (PAD). PADs can be divided into agammaglobulinemias, defects of class switch recombination, and hypogammaglobulinemias. Hypogammaglobulinemia is by far the most common entity, comprising nearly half of all PID diagnoses (2, 4). In specialized centers, common variable immunodeficiency disorder (CVID) is the most common form of hypogammaglobulinemia seen (estimated prevalence in the population 1:10,000–50,000) (5, 6). In the ESID Registry, CVID is strictly defined: age >4 years, markedly decreased serum IgG and IgA with or without low IgM, poor antibody response to vaccines, and exclusion of an underlying cause (http://www.esid.org). Many more patients live with less well described and understood forms of hypogammaglobulinemia: deficiency of IgG, IgG-subclass(es), IgM, IgA, and/or specific antibodies, alone, or in combination(s) (4). We refer to these forms as *unclassified* primary antibody deficiency (unPAD). Because of the moderately decreased immunoglobulin levels, unPADs are generally considered to be clinically milder. However, data regarding the clinical presentation, prognosis and treatment of unPAD patients are limited. These patients generally do not visit physicians in specialized/tertiary centers, and are often not treated with the immunodeficiency taken into consideration.

The symptoms of patients with hypogammaglobulinemia can lead to decreased quality of life, increased loss of participation in society (school, work) and higher health care costs (7–12). These patients often go unrecognized, because the general public as well as most health care professionals, who are not specialized in immunodeficiency, do not consider potential PID in patients with recurrent “normal” infections (responding to regular treatment, and not caused by an unexpected or opportunistic pathogen). Their concomitant fatigue is often interpreted to be of psychosocial origin or labeled as chronic fatigue syndrome.

To improve awareness and early detection of PID in patients with recurrent normal infections, we developed a Care Pathway for suspected primary immunodeficiency in the Jeroen Bosch hospital (JBZ), a large teaching hospital (secondary center) in the southern part of the Netherlands. All patient data obtained in regular care in the Pathway were collected and stored electronically. In this report, we present a detailed analysis of the available clinical data from the patients diagnosed with PAD during the first 4 years of the Pathway who gave informed consent for using their data for this purpose; these were mostly unPAD patients.

## Materials and methods

### Study design

Data were prospectively collected, pseudonymized, and stored on a protected server using Research Manager software developed by Cloud9 Health Solutions (Deventer, the Netherlands) as part of a standardized, 1-day Care Pathway for suspected PID. Data were collected on all patients referred to the Care Pathway from the start in February 2012 to June 2016. The primary objective of this study was to describe the patients, with special focus on unPAD patients, for this project including “deficiency of specific IgG (specific antibody deficiency—SPAD),” “IgA with IgG subclass deficiency,” “isolated IgG subclass deficiency,” “selective IgM deficiency,” and “selective IgA deficiency.” The secondary objective was to analyze whether subgroups could be identified. Only participants who gave written informed consent were included in this study; the Medical Ethical Committee Brabant approved the study.

### The care pathway

Patients were referred to the Care Pathway when there was a suspicion of potential immunodeficiency. Upon referral, data were collected electronically on patient and family history, and previous medical information was requested. Based on this information, the immunologist (author De Vries) decided whether visiting the Care Pathway was indicated, based on the clinical presentations of PID as presented in the ESID diagnostic protocol (3, 13). Patients could be referred by general practitioners or by medical specialists (e.g., pulmonologists, internists, ENT-surgeons, dermatologists). Seventy-seven percent lived in the encashment area of the JBZ (320,000 people), the remaining patients were referred from other parts of the country.

The Care Pathway comprised a visit to an immunologist specialized in the field of PID (author De Vries), in addition to indicated laboratory and radiologic evaluations, and pulmonary function tests. After completion of the Care Pathway, each patient was evaluated in a multidisciplinary team, attended by the immunologist, a pulmonologist, an internist, and specialized nurse. The team formulated an advice for each individual patient on: (1) presence or absence of PID, (2) indication for treatment with immunoglobulin substitution and/or (change of) antimicrobial prophylaxis, (3) indication for investigation of family members, and (4) necessity of referral to a tertiary center.

### Assessments

All assessments were performed during regular, routine patient care and included online questionnaires to be completed by patients, laboratory tests, pulmonary function tests, and imaging. All patients recorded the following social, demographic, and clinical characteristics: age, gender, smoking habit, previous symptoms, prescribed therapy, family history, and highest education level. Health-related quality of life (HRQoL) was measured using the age specific TNO-AZL questionnaires: TNO-AZL Pre-school children's Quality of Life questionnaire (TAPQOL, *parents* for children aged 1–5 years), TNO-AZL Children's Quality of Life questionnaire (TACQOL, *parents* and *children* for children aged 6–15 and 8–15 years, respectively), or TNO-AZL questionnaire for adult's HRQoL (TAAQOL, ≥16 years) (14). Items inquire about the incidence of physical, psychological, or social problems on different domains and are scored on a 3- or 4-point Likert scale (TAPQOL and TACQOL “never/occasionally/often” a problem with …; TAAQOL “no/a little/some/a lot of” difficulty in …). If a problem/difficulty is reported, parents and children 8–15 years rate how the children felt at those times on a 4-point Likert scale (TAPQOL “well/not very well/unwell/very unwell;” TACQOL “fine/not so good/quite bad/bad”); children 16 years and over and adult subjects rate how much that problem bothered them on a 4-point Likert scale (TAAQOL “not at all/a little/quite a lot/very much”). Higher scores indicate a better HRQoL. For interpretation of the various laboratory tests, age-matched reference values were used. For interpretation of pneumococcal antibody responses laboratory specific reference values were used^1^. Analysis of B- and T-cell subpopulations were performed as described previously (15).The High-resolution CT (HRCT) scans of the thorax were scored by a thoracic radiologist according to the “Chest CT in ADS” criteria^2^. Finally, the immunologist scored the diagnosis, first clinical presentation (3, 13) and disease status of the patient.

### Statistical analysis

Continuous variables were summarized by mean and standard deviation (SD) when normally distributed, and otherwise by median and inter-quartile range (IQR). Categorical variables were summarized by number and percentage. The group of children with PAD was too small to perform statistical analyses.

The domain scores of the TAPQOL, TACQOL, and TAAQOL were computed using the SPSS syntax provided by the authors. To ensure similarity with the Care Pathway data, individuals outside the observed age range of the Care Pathway patients (21–77) were excluded from the adult reference data before analysis, leaving data from 4,120 subjects in the reference dataset. Analyses examining the associations between categorical variables in the adult patients were performed using Fisher's exact test. Analyses examining the differences in continuous measures in the adult patients were performed using the unpaired *t*-test when normally distributed, and otherwise by the Mann-Whitney test. Because of the great number of comparisons, only *p*-values < 0.01 were regarded to be statistically significant. The spearman's rank correlation coefficient test was used to examine the association between IgG subclass levels and pneumococcal vaccination response. The following cut-off values were used to describe ρ: 0.00–0.19 “very weak;” 0.20–0.39 “weak;” 0.40–0.59 “moderate;” 0.60–0.79 “strong;” and 0.80–1.0 “very strong.”

To analyze whether the adult patient group could be divided into subgroups, K-means clustering was applied creating between 2 and 5 clusters. Variables that exhibited highly positively skewed distributions were analyzed on the log scale. The Calinski/Harabasz pseudo-F index was calculated for each cluster solution; the solution with the largest value of this index indicated the most distinct clustering, and was chosen as the optimal solution. Next, the differences between clusters were examined, by comparing variables between the clusters.

The logistic regression model was used for evaluating the predictive effect of family history, TAAQOL domains and HRCT findings for the patient diagnosis, classified as either CVID (according to the ESID Registry working diagnosis criteria) (16) or unPAD. For factors where the diagnosis was the same for all patients in a category, it was not mathematically possible to perform logistic regression, and Fisher's exact test was used instead. Firstly, the association between each factor and the outcome was examined separately in a series of univariable analyses. Subsequently, the joint association of the factors and the outcome was examined in a multivariable analysis. To restrict the number of variables in the second stage of the analysis, only those factors with a univariable *p*-value of ≤ 0.10 were used for this stage. A backwards selection procedure was used to retain only the statistically significant variables in the final model. This involves omitting non-significant variables, one at a time, until only the significant variables remain.

## Results

### The care pathway

**Figure 1:**
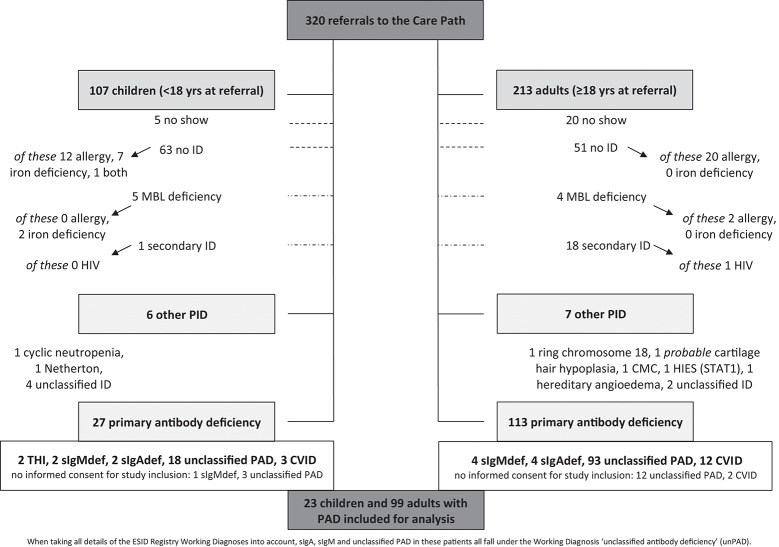
The population under study. Overview of all patients referred to the Care Pathway between February 2012 and June 2016 (inclusive) to answer the question “could this be primary immunodeficiency?” When taking all details of the ESID Registry Working Diagnoses into account, sIgAdef, sIgMdef, and unclassified PAD in these patients all fall under the Working Diagnosis “unclassified antibody deficiency” (unPAD) (16). Allergy, clinical symptoms, *and* proven sensitization by specific serum IgE and/or skin prick test; CVID, common variable immunodeficiency disorders (according to the ESID Registry working diagnoses); HIV, human immunodeficiency virus; Ig, immunoglobulin; iron deficiency, low iron stores, determined by serum ferritin level; MBL, mannose binding lectin; (other) (P)ID, (other) (primary) immunodeficiency (other meaning other than primary *antibody* deficiency); sIgAdef, selective IgA deficiency (according to the ESID Registry working diagnoses, but absence of *clinical* signs of T-cell deficiency was considered sufficient); sIgMdef, selective IgM deficiency (according to the ESID Registry working diagnoses, but absence of *clinical* signs of T-cell deficiency was considered sufficient); THI, definite transient hypogammaglobulinemia of infancy (the antibody deficiency has resolved during the period under study); unclassified ID *resp*. PAD, unclassified primary antibody *resp*. immuno-deficiency (according to the ESID Registry working diagnoses); yrs, years.

From the 320 patients that were referred to the Care Pathway between February 2012 and June 2016, 153 were shown to have some form of PID (92% PAD). In 99 adults and 23 children with PAD, written informed consent for inclusion in this analysis was obtained (details of patient selection process in Figure 1).

### Description of the pediatric patients

The group of 23 children with PAD was too small to perform any meaningful statistical analysis. An overview of their collected data is shown in Supplementary Table 1. The parents' main complaint at referral was that their child was “ill too often” in 70%, for 83% the main reason for referral was to find out the reason why (“what is the matter with him/her”). Fifteen children were referred by their pediatrician, four by their general practitioner, three by their ENT surgeon, and one by their dermatologist. Age at referral was 2–16 yrs (mean 7.5 yrs; median 6 yrs); boys predominated (74%). The clinical presentation (3, 13) was “recurrent ENT and airway infections” in 78%. Thirty-nine percent had an iron deficiency and 39% had an increased total IgE, one third of these had ≥1 positive specific IgE in their serum (tree pollen, house dust mite, cat dander, dog dander, and/or grass pollen). In 22%, other family member(s) also had a PAD diagnosis (already known in three, not yet known in two cases). A pulmonary HRCT scan was performed in five patients; two showed bronchopathy, none bronchiectasis. In 41%, a mild to moderate decrease in HRQoL was reported. Five were put on prophylactic co-trimoxazole, two on subcutaneous, and two on intravenous immunoglobulin substitution.

### Description of the adult patients

**Figure 2:**
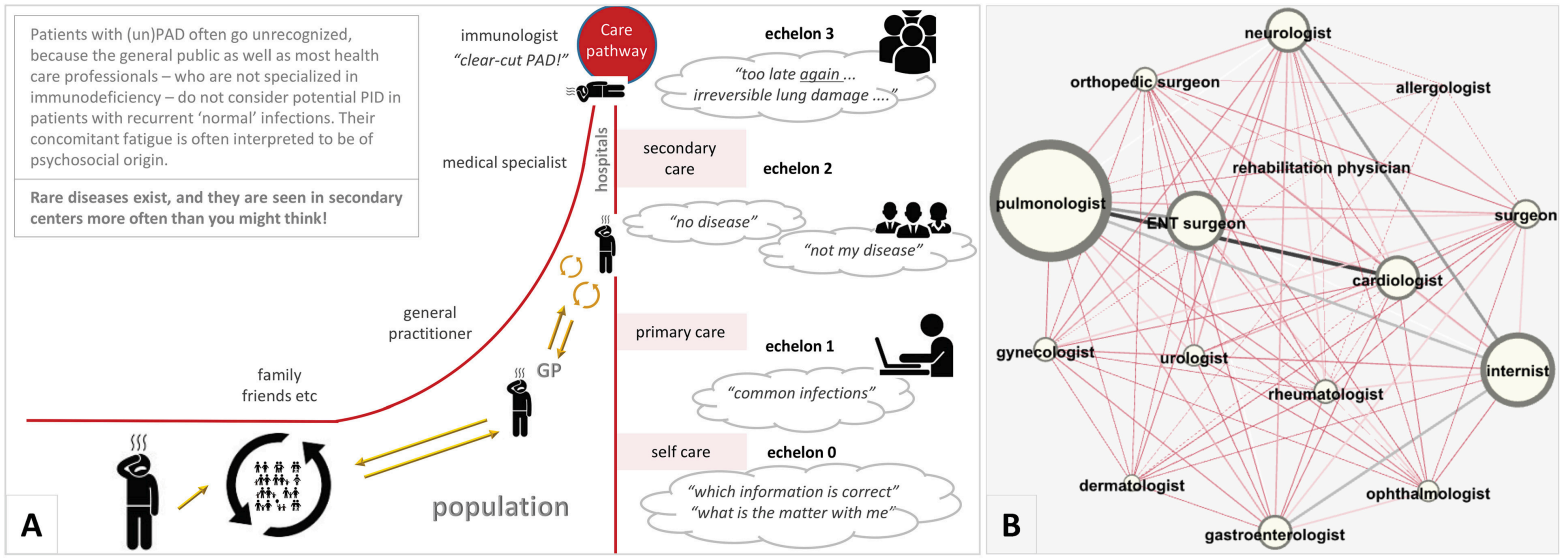
**(A)** The long journey patients with (un)PAD often face toward diagnosis. **(B)** The clinical specialists the 99 adult patients with PAD had visited prior to their referral to the Care Pathway. The size of the nodes indicates the number of times the patients encountered that specialism and the size of the connecting lines between two nodes indicates the number of times that patients were known to both specialisms (created by Gephi software). ENT, ear-nose-throat specialist; GP, general practitioner; PAD, primary antibody deficiency.

Of the 99 PAD patients (71 women, 72%), 89 had unPAD (66 women, 74%), and 10 had CVID (5 women, 50%) according to the working diagnoses used in the ESID online Registry (16). An overview of their collected data is shown in Supplementary Table 2. Age at referral was 21.0–77.4 yrs (mean 51.3 yrs; median 51.5 yrs). BMI, smoking habits, and highest educational level were comparable with the Dutch LISS panel, a representative sample of the Dutch population (http://www.lissdata.nl). The majority (*n* = 43) was referred by their pulmonologist, followed by their general practitioner (*n* = 35), and internist (*n* = 15). The wide variety of clinical specialists the patients with PAD had visited prior to their referral to the Care Pathway is illustrated in Figure 2. The main complaint at referral was “ill too often” in 87, with airway infections in 35, chronic cough in 4, and ENT-infections in 9 patients. For 35 patients being extremely tired and having no energy was their most important complaint. The immunologist characterized the initial presentation as “recurrent ENT and airway infections” in 88, “auto-immune or chronic inflammatory disease; lymphoproliferation” in 10, and “unusual infections or unusually severe course of infections” in 1 of the patients (13).

**Figure 3:**
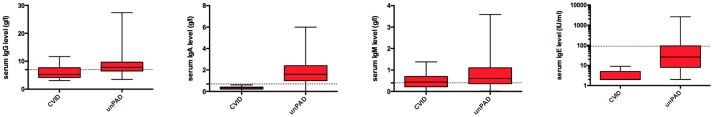
Mean serum immunoglobulins at diagnosis of the adults with CVID vs. unPAD. CVID, common variable immunodeficiency disorder; unPAD, unclassified primary antibody deficiency (17).

**Figure 4:**
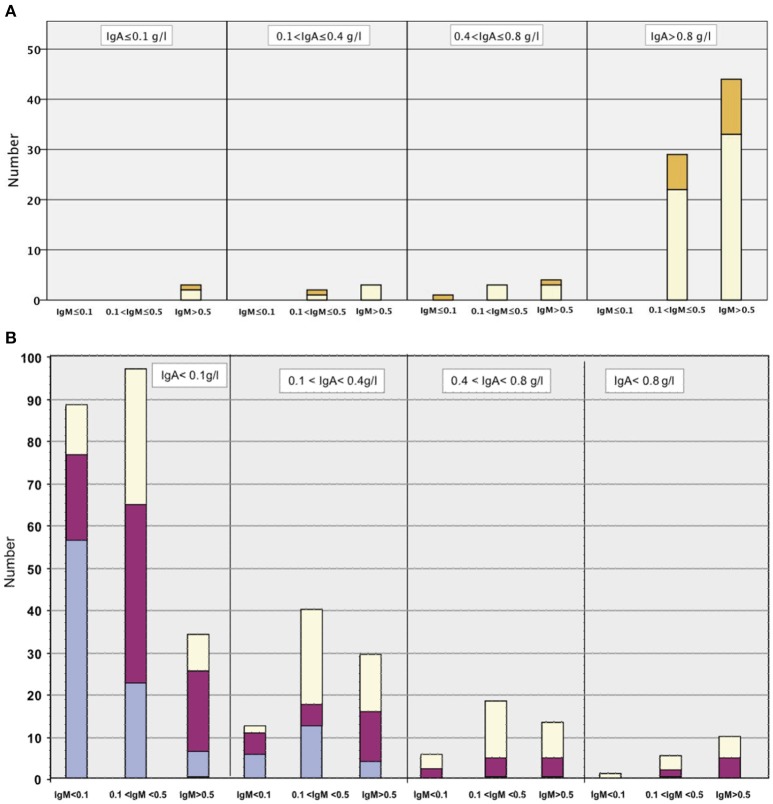
**(A)** unPAD cohort (89 patients): serum immunoglobulins (g/l) at diagnosis. Each column is divided into four parts, depending on the IgG levels; those in the light purple section with IgG ≤ 1.0 g/l; those in the dark purple section with IgG>1.0 ≤ 3.0 g/l; those in the yellow section with IgG >3.0 ≤ 6.5 g/l and those in the orange section with IgG > 6.5 g/l. **(B)** For comparison: European CVID cohort (Chapel et al., 334 patients): serum immunoglobulins (g/l) at diagnosis. Unabridged from Chapel et al. (18). Permission was obtained from the editor in chief of Blood to use the previously published **(B)**.

As expected by the clinical definition of CVID and unPAD, the median immunoglobulin levels at diagnosis were lower in patients with CVID compared to unPAD patients (Figure 3). The high prevalence of undetectable serum IgE in the CVID patients (57%, 4/7 patients) is in agreement with the recently published large CVID cohort by Lawrence et al. (17), demonstrating that low/undetectable serum IgE is characteristic of CVID. Similar to the previously published European CVID cohort by Chapel et al. (18), the 89 unPAD patients were divided into categories based on presenting serum immunoglobulin levels (Figure 4). In most unPAD patients (62%) IgG and IgA levels were between 3.1 and 6.5 and > 0.8 g/l, respectively, while in the cohort of Chapel et al. the majority of patients (94.2%) had initial IgG levels < 4.5 g/l at diagnosis (18). Classification of the unPAD patients according to their immunoglobulin levels and pneumococcal vaccination response (PVR) is shown in Supplementary Figures 1, 2. Median B and T lymphocyte counts were largely within the normal range (Table 1). A pulmonary HRCT scan was performed in 60 patients; of these, 53 had unPAD. Of the unPAD patients, 25 (47%) showed bronchial wall thickening, 24 (44%) bronchiectasis in one or more lobe(s), 11 (21%) central or peripheral mucus plugging, and 10 (19%) atelectasis. Twenty patients were put on prophylactic co-trimoxazole (CVID, *n* = 1; unPAD, *n* = 19), 6 on subcutaneous and 23 on intravenous immunoglobulin substitution (CVID, *n* = 9; unPAD, *n* = 20).

**Table 1:** Absolute number of lymphocyte subpopulations in peripheral blood in the unPAD patients.

**Population**	***N* =**	**Reference range × 10^e^9/l^a^**	**Median (IQR) × 10^e^9/l**	**Decreased (*n*)**
Helper T-lymphocytes (CD3^+^CD4^+^)	75	0.5–2.0	0.9 (0.7–1.2)	4
T-lymphocytes (CD3^+^)	75	0.78–3.0	1.4 (1.2–2.0)	4
Cytotoxic T-lymphocytes (CD3^+^CD8^+^)	75	0.2–1.2	0.5 (0.3–0.7)	3
NK-cells (CD3^+^CD16^+^and/orCD56^+^)	74	0.10–1.2	0.2 (0.1–0.3)	4
B-lymphocytes (CD19^+^)	75	0.064–0.82	0.20 (0.11–0.30)	3
IgM only memory B-lymphocytes (CD19^+^CD27^+^IgM^+^IgD^−^)	67	0.0011–0.015	0.0034 (0.0013–0.0066)	11
Switched memory B-lymphocytes (CD19^+^CD27^+^IgM^−^IgD^−^)	67	0.0045–0.13	0.021 (0.011–0.044)	6
CD21low B cells (CD19^+^CD21^low^CD38^low^)	67	0.0017–0.049	0.0058 (0.003–0.010)	7^b^
Naïve B-lymphocytes (CD19^+^CD27^−^IgM^+^IgD^+^)	67	0.028–0.55	0.09 (0.046–0.16)	10
Transitional B cells (CD19^+^CD38^++^IgM^++^)	67	0.0006–0.10	0.0082 (0.0031–0.016)	7

a *From Schatorjé et al. (15)*.

b *Increased CD21^low^ cell population in 2 patients*.

### Statistical analyses in the adult patients

**Figure 5:**
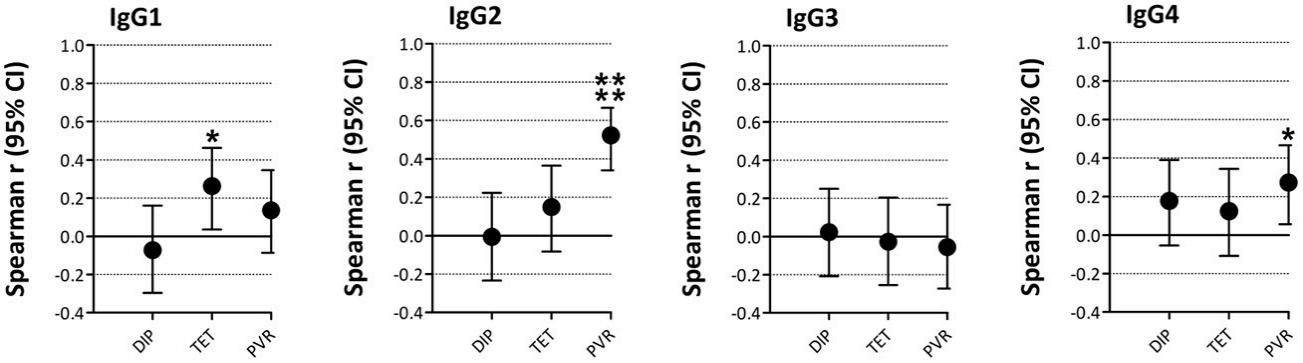
Correlations between vaccination responses and IgG-subclass levels. The graphs show the Spearman's correlation coefficients and 95% confidence interval (CI) for IgG1-4 vs. vaccination responses. *p < 0.05, **p < 0.01, ***p < 0.001, ****p < 0.0001. DIP, diphtheria; TET, tetanus; PVR, pneumococcal vaccination response.

We assessed whether the concentrations of individual IgGsc correlated with the response to specific vaccine challenges (Figure 5). Spearman's correlations between IgGsc and PVR were moderate for IgG2 (ρ 0.52, 95% confidence interval (CI) 0.34–0.67, *p* < 0.0001), and weak for IgG4 (ρ 0.27, 95% CI 0.06–0.47, *p* < 0.05). There was a weak correlation between IgG1 and antigen-specific antibody response against tetanus toxoid (ρ 0.26, 95% CI 0.04–0.46, *p* < 0.05). Detailed results of all IgG subclasses plotted on a logarithmic scale with each PP serotype are shown in Supplementary Figures 3, 4.

The combination of PVR, IgA, and IgG at first presentation to the Pathway could not predict bronchiectasis in the adult PAD patients. Pulmonary HRCT scan findings were also not associated with referring doctor type, gender, the complaint “being always ill,” CVID vs. unPAD diagnosis, or type of prescribed therapy. Mucus plugging occurred significantly more often in patients with “recurrent ENT and airway infections” and/or chronic cough (41%), compared to patients who were always tired or had other complaints (15%) (Fisher exact test [98], *p* = 0.04).

**Figure 6:**
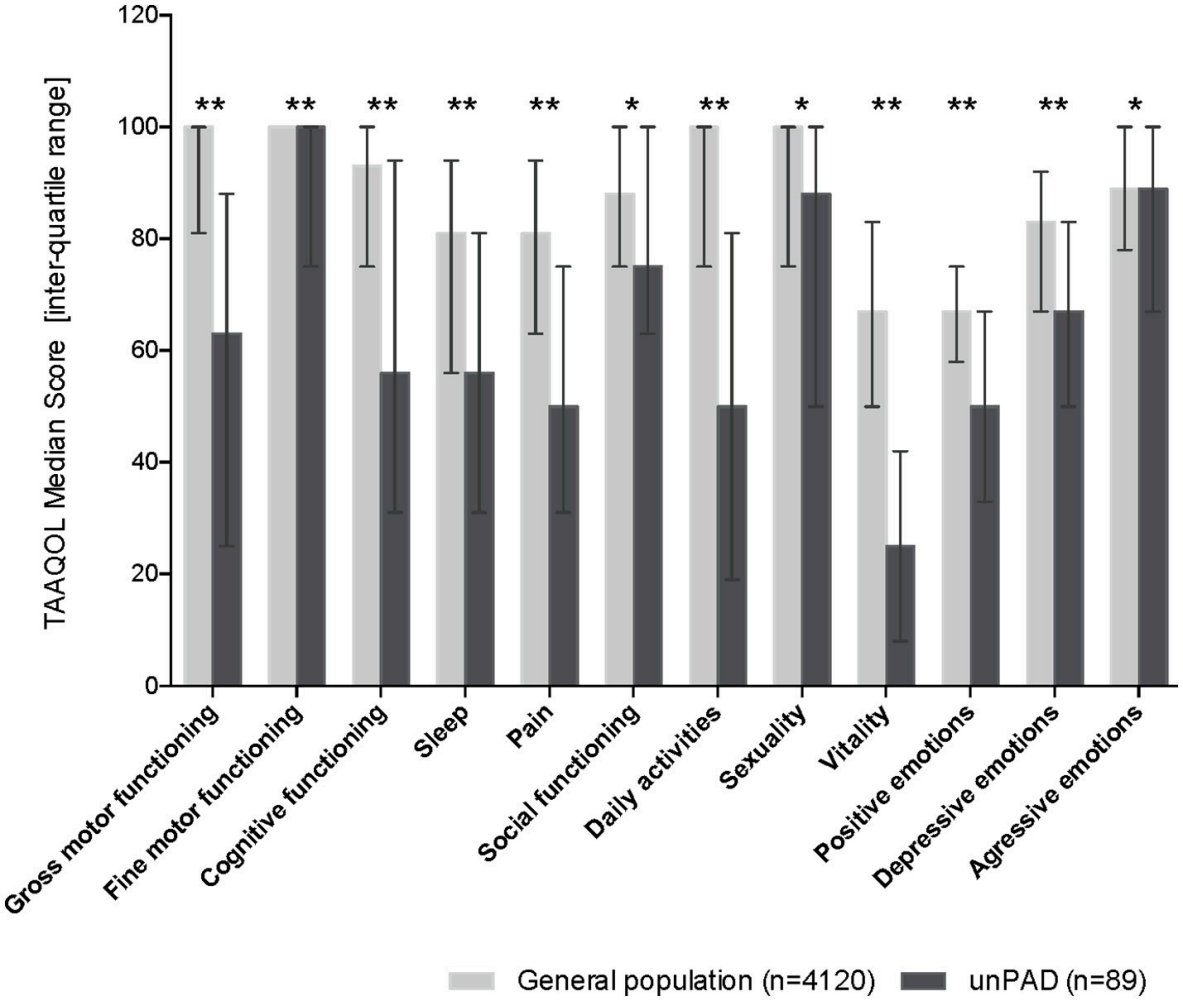
HR-QoL in 89 unPAD patients compared to the age-matched subjects from the reference dataset (*n* = 4,120). Higher scores indicate better HR-QoL (TAAQOL); the maximum score is 100. Error bars indicate inter-quartile ratios. Statistically significant differences are indicated by an asterisk: *p < 0.01; **p < 0.001. HR-QoL, health-related quality of life; TAAQOL, TNO-AZL questionnaire for adult's HRQoL; unPAD, unclassified primary antibody deficiency.

Nine CVID and Eighty nine unPAD patients completed the online TAAQOL questionnaire. Patients with unPAD scored significantly worse (*P* < 0.01) on all domains compared to the subjects in the reference dataset (Figure 6). 3/9 CVID patients had already started immunoglobulin substitution when completing the TAAQOL questionnaire, therefore, it is impossible to draw conclusions from this group. In order to create separate subgroups of similar patients, associations between the key variables were examined. Cluster analysis showed that specifying two clusters resulted in the highest *F*-statistic (24.8). The two clusters varied significantly for all but fine motor functioning TAAQOL domain scores, but these clusters did not match the division between CVID and unPAD patients. The full results of the performed analyses can be found in Supplementary Tables 3, 4.

## Discussion

This description of patients with primary antibody deficiency (PAD) is unique in its secondary center population and its focus on unPAD. It is also the first study to examine HRQoL in unPAD patients. Our results demonstrate that unPAD can result in severe patient burden. Besides the high proportion of patients suffering from bronchiectasis, their HRQoL was significantly impaired in all domains compared to the reference population. This study shows for the first time that many unPAD patients had to cope simultaneously with pain, negative feelings and impairments in cognition, home management tasks, sleep, social interaction, and work. A small number of previous studies have investigated HRQoL in PADs (7, 9, 19), some focusing solely on CVID (11, 12, 20, 21), but patients with unPAD were not included in these studies. This unPAD group, generally considered to be a “mild” form of hypogammaglobulinemia, has hardly received any attention in the literature (22). Based on our results, we strongly recommend to change this.

The most severely affected HRQoL domain was vitality, indicating unPAD patients feel extremely tired and worn out. This parallels the above-average observed frequency of fatigue in PAD patients, not only compared to the general population, but also compared to the total PID population (23). Fatigue is an important and debilitating problem, because it can lead to decreased daily activities, resulting in general deconditioning, which further affects fatigue and HRQoL in general. Clinicians should be aware of this.

While it was previously thought that bronchiectasis is the result of repeated infections due to deficient antibody production (24), there is increasing evidence that immune dysregulation plays an important role in the disease process (25). Based on these new insights, the high proportion of unPAD patients suffering from bronchiectasis at presentation in our cohort (44%)—similar to the frequency reported in the literature for CVID patients (22, 26–29)—is not that surprising. Clearly, despite the only moderately decreased immunoglobulin levels in unPAD compared to CVID patients, unPAD can result in comparable serious pulmonary complications.

Interestingly, there were nearly twice as many boys with unPAD in our pediatric cohort, but considerably more women than men in the adult group (74%). This may indicate that these diseases differ in different age groups. Perhaps unknown X-linked disease plays a role in some of these boys with antibody deficiency (30, 31). The female predominance in the adult unPAD patients suggests the pathogenesis of unPAD may differ between adult women and men. The tendency for immune dysregulation is widely acknowledged to be greater in women (32), but it is also possible that there are protective factors in men. It would be interesting to confirm this pattern in a much larger cohort, and to further explore potential gender-specific mechanisms.

IgG-subclass measurements could not predict pneumococcal vaccination responses (PVRs), or vice versa. Only a moderate correlation between IgG2-subclasses and PVRs was found. These results are in agreement with previous studies in patients with Hodgkin's lymphoma (33), in children with chronic chest infections (34), and in patients with IgA deficiency (35). Thus, both are needed to fully explore the immune status of an individual patient.

Our study has several limitations. First, the sample size was limited. Therefore, the insignificant results of the exploration of distinct subgroups in the unPAD cohort or differences between the unPAD and CVID patients, might be caused by the limited detection power. A future study in a much larger cohort may well be able to reveal separate clinical entities; the ESID online Registry would be a good tool for this. Second, the few CVID patients had partly already started their immunoglobulin substitution therapy. This means our study should be mainly used as a thorough description of the—to date—largest cohort of unPAD patients, not as an important source for comparison of unPAD with CVID. Third, genetic testing was not performed in most patients; it would be interesting to investigate this. It is possible that mildly affected patients with a known genetic defect are “hidden” in this cohort. Despite these limitations, our data show, contrary to what is currently assumed by most immunologists in specialized/tertiary centers, that “mild” hypogammaglobulinemia can be a serious condition. Also for those patients, early detection and adequate treatment is important.

## Author contributions

LJ and EdV designed the study and wrote the manuscript. EdV acquired the data. PB carried out statistical analyses. JdV helped with the interpretation of the quality of life data. AK scored the HRCT scans of the thorax according to the Chest CT in ADS criteria. TM, HP, JvE, MS, and AP reviewed the results and contributed to the final version of the manuscript. MS and AP analyzed associations between IgG subclass levels and pneumococcal vaccination responses.

### Conflict of interest statement

The authors declare that the research was conducted in the absence of any commercial or financial relationships that could be construed as a potential conflict of interest.

## Footnotes

^1^www.umcutrecht.nl/subsite/medische-immunologie/diagnostiek

^2^www.chest-ct-group.eu

## Supplementary material

Supplementary Figure 1 Division into subgroups according to immunoglobulin levels and pneumococcal vaccination responses of the adult unPAD cohort. CVID, common variable immunodeficiency disorder; IgGsc, IgG-subclass(es); PVR, pneumococcal vaccination response; SPAD, specific antibody deficiency; unPAD, unclassified primary antibody deficiency.

Supplementary Figure 2 Relation between vaccination responses and IgG-subclass levels. The bar graphs display the percentage of low IgG1, IgG2, IgG3, and IgG4, respectively between **(A)**. normal vaccination response, and **(B)**. abnormal vaccination response.

Supplementary Figure 3 Post-vaccination specific antibody titers against separate pneumococcal serotypes plotted against IgG1, IgG2 on a logarithmic scale. Per separate graph, patients were classified using the cut-off values for IgG-subclasses and vaccine responses to pneumococcal serotypes (dotted lines in the graphs). To be able to display patients' data points into the graphs, a pneumococcal serotype value of 0 g/L was changed to “0.01” g/L, which did not influence the classification.

Supplementary Figure 4 Post-vaccination specific antibody titers against separate pneumococcal serotypes plotted against IgG3, and IgG4 on a logarithmic scale. Per separate graph, patients were classified using the cut-off values for IgG-subclasses and vaccine responses to pneumococcal serotypes (dotted lines in the graphs). To be able to display patients' data points into the graphs, a pneumococcal serotype value of 0 g/L was changed to “0.01” g/L, which did not influence the classification.

Supplementary Table 1 The children with primary antibody deficiency. Comprehensive overview of clinical and laboratory data of the 23 children (< 18yrs at referral) with PAD who visited the Care Path February 2012 - June 2016 (inclusive) and for whom informed consent for inclusion in this study was obtained.

Supplementary Table 2 Descriptive statistics in 99 adults with primary antibody deficiency.

Supplementary Table 3 Statistical analyses in 99 adults with primary antibody deficiency.

Supplementary Table 4 Clustering analyses in 99 adults with primary antibody deficiency.

## Abbreviations

BMI: body mass index
CVID: common variable immunodeficiency disorders
ENT: ear nose throat
ESID: European Society for Immunodeficiencies
HRCT: high resolution computerized tomography
HRQoL: health-related quality of life
IgGsc: IgG-subclass
IQR: inter-quartile range
JBZ: Jeroen Bosch hospital
yrs: years
LISS: Longitudinal Internet Studies for the Social sciences
PAD: predominantly (primary) antibody deficiency
PID: primary immunodeficiency
PP: pneumococcal polysaccharide
PVR: pneumococcal vaccination response
SD: standard deviation
TAAQOL: TNO-AZL questionnaire for adult's HRQoL (≥16 years of age)
TACQOL: TNO-AZL questionnaire for children's HRQoL (6–15 years of age)
TAPQOL: TNO-AZL questionnaire for pre-school children's HRQoL (1–5 years of age)
unPAD: unclassified primary antibody deficiency.
