# Supplementary figures and images for: Mild Hypogammaglobulinemia Can Be a Serious Condition
Source: Front Immunol. 2018 Oct 15;9:2384. doi: 10.3389/fimmu.2018.02384 (PMC6196282; doi:10.3389/fimmu.2018.02384)

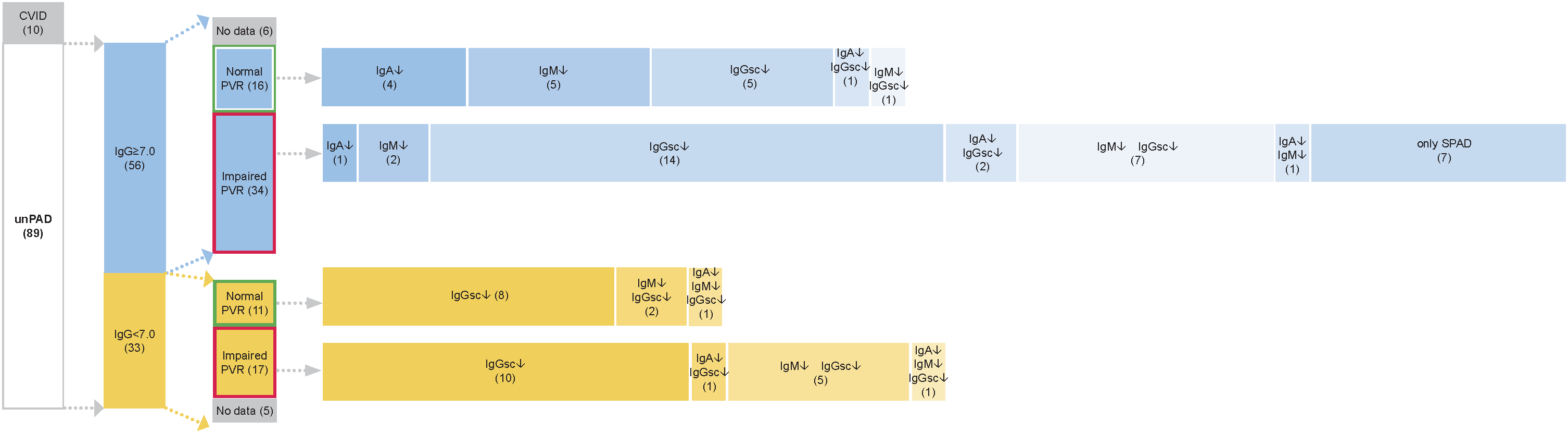

Supplement: Supplementary Figure 1 — Division into subgroups according to immunoglobulin levels and pneumococcal vaccination responses of the adult unPAD cohort. CVID, common variable immunodeficiency disorder; IgGsc, IgG-subclass(es); PVR, pneumococcal vaccination response; SPAD, specific antibody deficiency; unPAD, unclassified primary antibody deficiency. [file Image_1.TIFF]

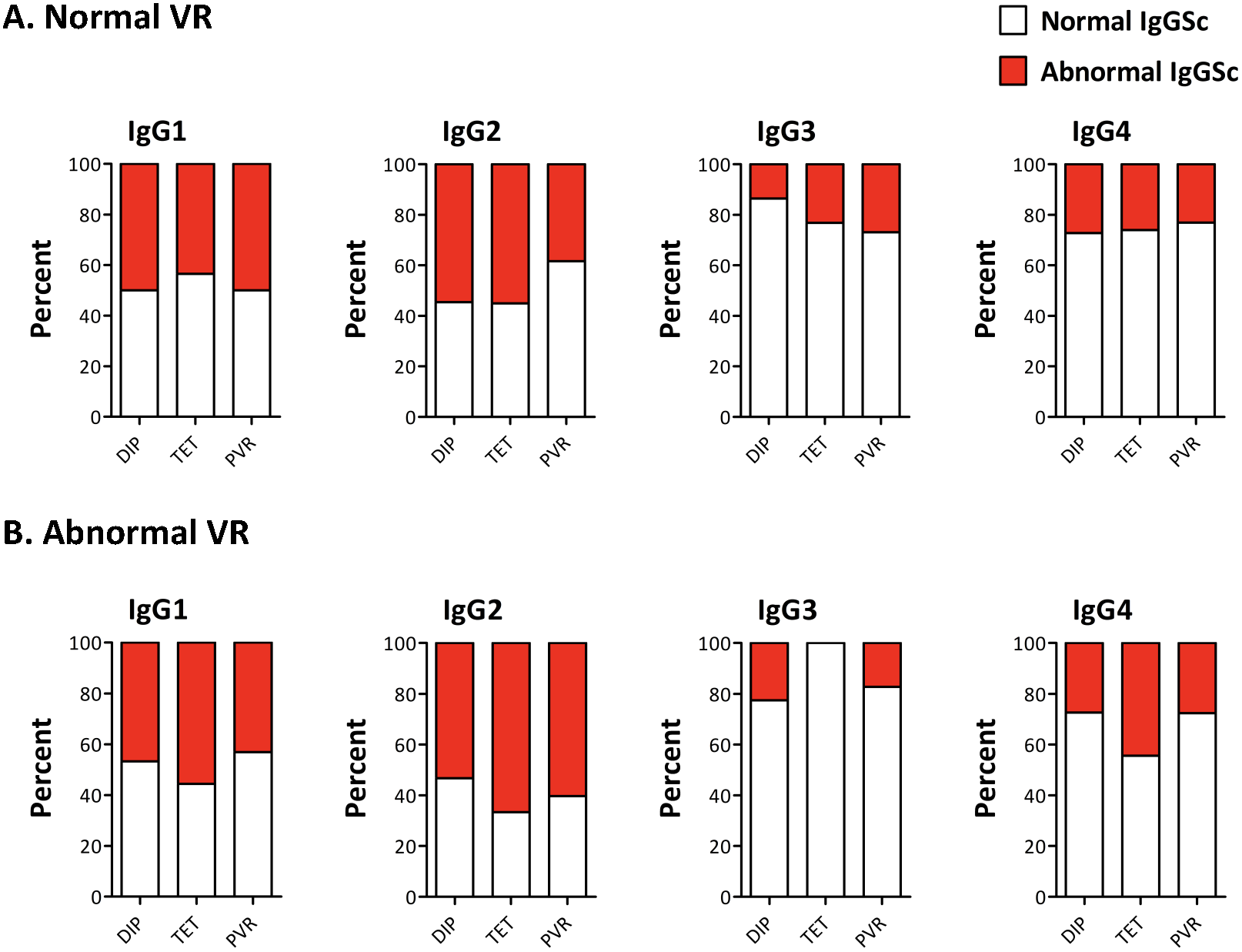

Supplement: Supplementary Figure 2 — Relation between vaccination responses and IgG-subclass levels. The bar graphs display the percentage of low IgG1, IgG2, IgG3, and IgG4, respectively between (A). normal vaccination response, and (B). abnormal vaccination response. [file Image_2.TIFF]

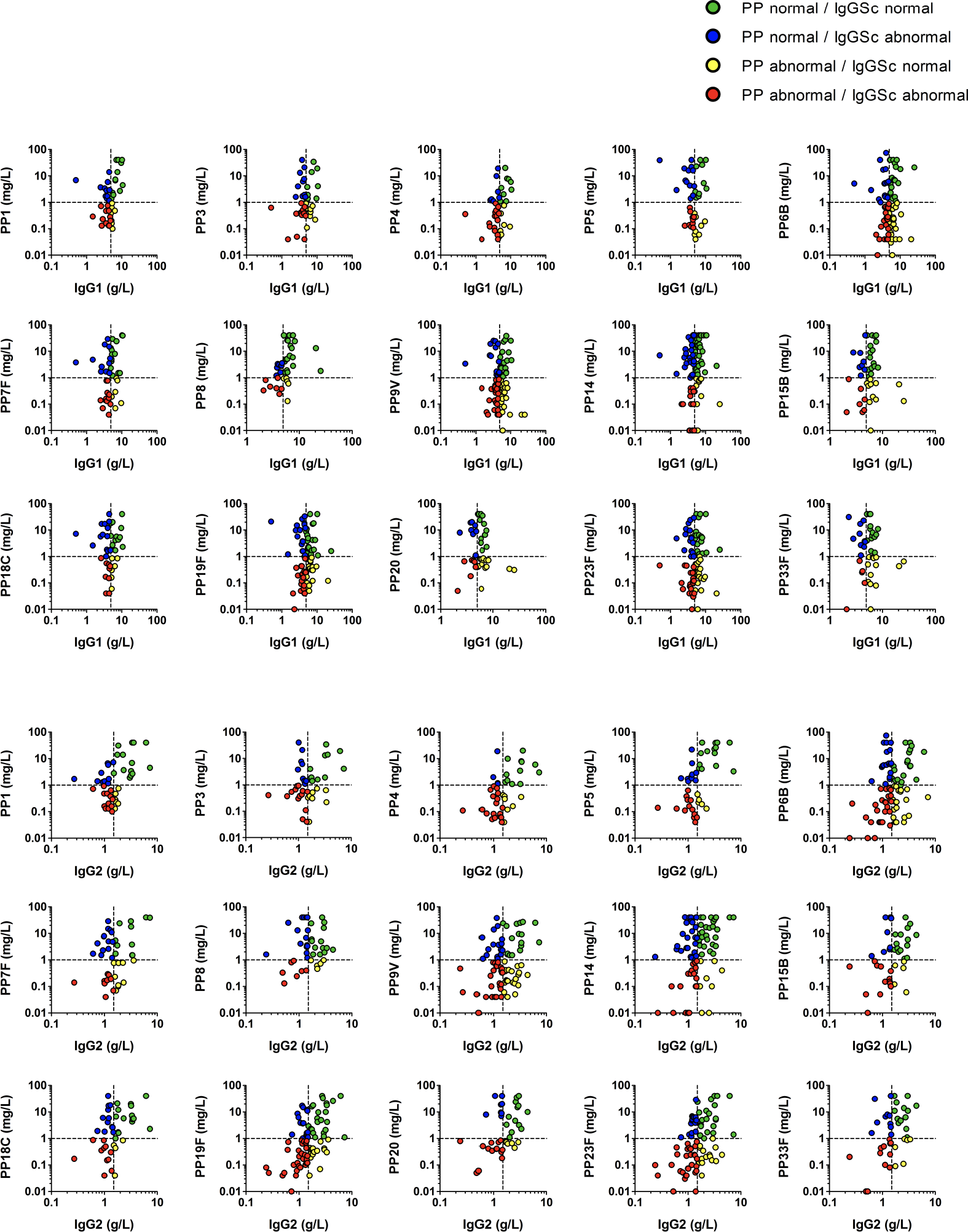

Supplement: Supplementary Figure 3 — Post-vaccination specific antibody titers against separate pneumococcal serotypes plotted against IgG1, IgG2 on a logarithmic scale. Per separate graph, patients were classified using the cut-off values for IgG-subclasses and vaccine responses to pneumococcal serotypes (dotted lines in the graphs). To be able to display patients' data points into the graphs, a pneumococcal serotype value of 0 g/L was changed to “0.01” g/L, which did not influence the classification. [file Image_3.TIFF]

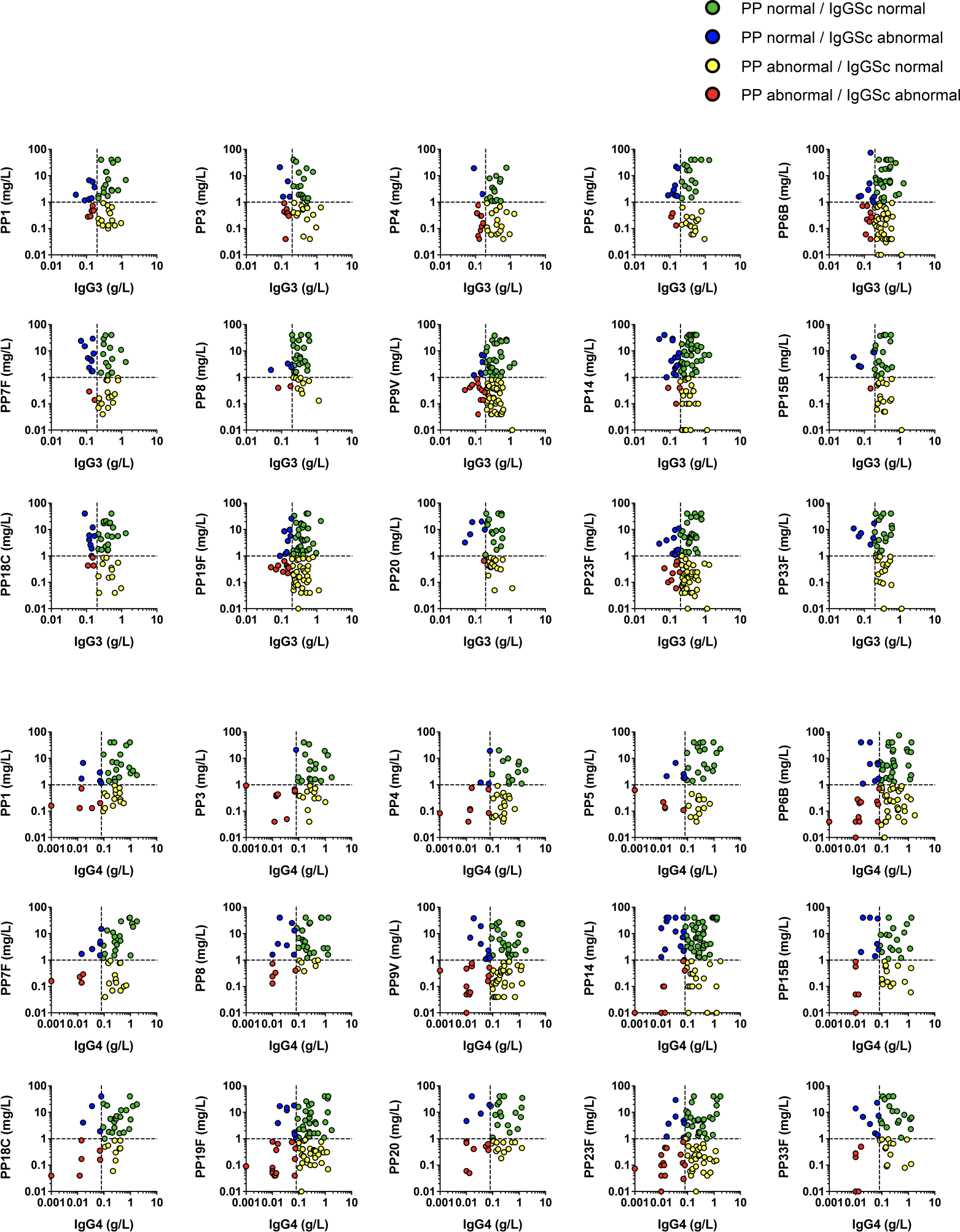

Supplement: Supplementary Figure 4 — Post-vaccination specific antibody titers against separate pneumococcal serotypes plotted against IgG3, and IgG4 on a logarithmic scale. Per separate graph, patients were classified using the cut-off values for IgG-subclasses and vaccine responses to pneumococcal serotypes (dotted lines in the graphs). To be able to display patients' data points into the graphs, a pneumococcal serotype value of 0 g/L was changed to “0.01” g/L, which did not influence the classification. [file Image_4.TIFF]
