# Supplementary material for: Mild Hypogammaglobulinemia Can Be a Serious Condition
Source: Front Immunol. 2018 Oct 15;9:2384. doi: 10.3389/fimmu.2018.02384 (PMC6196282; doi:10.3389/fimmu.2018.02384)
Supplement: Supplementary Table 1 — The children with primary antibody deficiency. Comprehensive overview of clinical and laboratory data of the 23 children (< 18yrs at referral) with PAD who visited the Care Path February 2012 - June 2016 (inclusive) and for whom informed consent for inclusion in this study was obtained. [file Table_1.docx]

**Supplementary table 1. The children with primary antibody deficiency.** Comprehensive overview of clinical and laboratory data of the 23 children (<18yrs at referral) with PAD who visited the Care Path February 2012 - June 2016 (inclusive) and for whom informed consent for inclusion in this study was obtained.

| ***= THE CHILDREN =*** | **C1** | **C2** | **C3** | **C4** | **C5** | **C6** | **C7** | **C8** | **C9** | **C10** | **C11** | **C12** | **C13** | **C14** | **C15** | **C16** | **C17** | **C18** | **C19** | **C20** | **C21** | **C22** | **C23** |
| --- | --- | --- | --- | --- | --- | --- | --- | --- | --- | --- | --- | --- | --- | --- | --- | --- | --- | --- | --- | --- | --- | --- | --- |
| gender | M | M | M | M | F | F | M | M | M | M | M | M | M | F | M | M | M | M | F | F | M | F | M |
| age at referral (yrs) | 13 | 13 | 13 | 13 | 10 | 9 | 8 | 9 | 7 | 7 | 6 | 6 | 6 | 4 | 5 | 6 | 4 | 16 | 4 | 3 | 2 | 5 | 3 |
| first clinical presentation^1^ | res | res | res | res | res | res | res | ftt | pyo | res | res | res | res | un | res | ftt | ail | res | res | res | res | res | res |
| ESID Registry working diagnosis^2^ | cvid | uAD | uAD | uAD | cvid | cvid | uAD | sIgM | uAD | uAD | uAD | uAD | uAD | uAD | THI | uAD | uAD | sIgA | uAD | THI | uAD | sIgA | uAD |
| additional diagnostic information | iron | - | - | - | - | - | iron | eci | eci | iron | - | iron | iron | shingles | iron | iron | cITP  MBL | - | iron | BHR | - | - | iron |
| familial case | N | Y | N | N | Y | Y | Y | N | N | N | N | N | N | N | N | N | N | N | N | N | N | Y | N |
| HRCT scan lungs^3^ | nml | brp | - | - | brp | nml | - | - | - | nml | - | - | - | - | - | - | - | - | - | - | - | - | - |
| therapy (related to ID) | sc | - | - | ab | sc | iv | - | - | - | iv | - | fe | ab | - | fe | fe | - | - | fe | ab | - | ab | ab |
| IgG (g/l)^4^ | 5.8  (5,2-15,6) | 4.9  (5,2-15,6) | 10.9  (5,2-15,6) | 8.1  (5,2-15,6) | 5.1  (5,2-15,6) | 4.4  (5,2-15,6) | 7.2  (5,2-15,6) | 9.9  (5,2-15,6) | 9.1  (5,2-15,6) | 3.6  (5,2-15,6) | 11.4  (4,3-13,4) | 5.0  (4,3-13,4) | 9.0  (4,3-13,4) | 6.8  (4,3-13,4) | 5.2  (4,3-13,4) | 8.3  (4,3-13,4) | 5.6  (4,3-13,4) | 12.8  (7,0-16,0) | 4.5  (4,3-13,4) | 5.2  (4,3-13,4) | 6.4  (4,3-13,4) | 16.0  (4,3-13,4) | 8.2  (4,3-13,4) |
| IgG1 (g/l) ^4^ | 5.3  (3,7-12,8) | 2.8  (3,7-12,8) | 8.1  (3,7-12,8) | 6.1  (3,7-12,8) | 3.8  (3,7-12,8) | 2.8  (3,7-12,8) | 5.1  (3,7-12,8) | - | 6.8  (3,7-12,8) | 2,8  (3,7-12,8) | 9.3  (3,2-10,0) | 4.3  (3,2-10,0) | 7.0  (3,2-10,0) | 5.2  (3,2-10,0) | 3.7  (3,2-10,0) | 7.4  (3,2-10,0) | 4.5  (3,2-10,0) | 8.5  (3,7-12,8) | 3.9  (3,2-10,0) | 3.4  (3,2-10,0) | 5.1  (3,2-10,0) | 11.4  (3,2-10,0) | 6.5  (3,2-10,0) |
| IgG2 (g/l) ^4^ | 0.40  (0,85-6,1) | 1.70  (0,85-6,1) | 1.07  (0,85-6,1) | 0.84  (0,85-6,1) | 0.96  (0,85-6,1) | 0.98  (0,85-6,1) | 1.25  (0,85-6,1) | - | 1.28  (0,85-6,1) | 0.76  (0,85-6,1) | 0.57  (0,52-3,4) | 0.79  (0,52-3,4) | 0.57  (0,52-3,4) | 0.62  (0,52-3,4) | 0.68  (0,52-3,4) | 0.59  (0,52-3,4) | 0.65  (0,52-3,4) | 2.12  (0,85-6,1) | 0.14  (0,52-3,4) | 1.44  (0,52-3,4) | 0.77  (0,52-3,4) | 3.57  (0,52-3,4) | 0.93  (0,52-3,4) |
| IgG3 (g/l) ^4^ | 0.16  (0,13-1,63) | 0.74  (0,13-1,63) | 0.16  (0,13-1,63) | 0.61  (0,13-1,63) | 0.68  (0,13-1,63) | 0.21  (0,13-1,63) | 0.22  (0,13-1,63) | - | 0.12  (0,13-1,63) | 0.19  (0,13-1,63) | 0.81  (0,13-1,33) | 0.23  (0,13-1,33) | 0.22  (0,13-1,33) | 0.15  (0,13-1,33) | 0.17  (0,13-1,33) | 0.20  (0,13-1,33) | 0.25  (0,13-1,33) | 0.50  (0,13-1,63) | 0.04  (0,13-1,33) | 0.22  (0,13-1,33) | 0.14  (0,13-1,33) | 0.80  (0,13-1,33) | 0.49  (0,13-1,33) |
| IgG4 (g/l) ^4^ | 0.029  (0,023-2,3) | 0.235  (0,023-2,3) | 0.146  (0,023-2,3) | 0.012  (0,023-2,3) | 0.221  (0,023-2,3) | 0.019  (0,023-2,3) | 0.419  (0,023-2,3) | - | 0.062  (0,023-2,3) | 0.065  (0,023-2,3) | 0.367  (0,012-1,58) | 0.039  (0,012-1,58) | 0.065  (0,012-1,58) | 0.160  (0,012-1,58) | 0.526  (0,012-1,58) | 0.028  (0,012-1,58) | 0.014  (0,012-1,58) | 0.228  (0,023-2,3) | 0.044  (0,012-1,58) | 0.171  (0,012-1,58) | 0.105  (0,012-1,58) | 0.668  (0,012-1,58) | 0.073  (0,012-1,58) |
| IgA (g/l) ^4^ | 0.50  (0,54-3,6) | 0.83  (0,54-3,6) | 1.07  (0,54-3,6) | 2.35  (0,54-3,6) | 0.28  (0,54-3,6) | 0.28  (0,54-3,6) | 0.08  (0,54-3,6) | 1.28  (0,54-3,6) | 0.92  (0,54-3,6) | 0.61  (0,54-3,6) | 1.00  (0,19-2,2) | 0.31  (0,19-2,2) | 0.89  (0,19-2,2) | 0.52  (0,19-2,2) | 0.86  (0,19-2,2) | 1.29  (0,19-2,2) | 0.67  (0,19-2,2) | 0.25  (0,70-4,0) | 0.40  (0,19-2,2) | 0.25  (0,19-2,2) | 0.45  (0,19-2,2) | 0.00  (0,19-2,2) | 0.44  (0,19-2,2) |
| IgM (g/l) ^4^ | 0.21  (0,31-2,4) | 0.39  (0,31-2,4) | 0.79  (0,31-2,4) | 0.83  (0,31-2,4) | 0.57  (0,31-2,4) | 0.47  (0,31-2,4) | 0.73  (0,31-2,4) | 0.22  (0,31-2,4) | 0.87  (0,31-2,4) | 0.99  (0,31-2,4) | 0.95  (0,21-1,8) | 0.61  (0,21-1,8) | 1.15  (0,21-1,8) | 0.52  (0,21-1,8) | 0.77  (0,21-1,8) | 1.63  (0,21-1,8) | 0.63  (0,21-1,8) | 0.44  (0,40-2,3) | 0.27  (0,21-1,8) | 0.20  (0,21-1,8) | 0.42  (0,21-1,8) | 0.83  (0,21-1,8) | 1.20  (0,21-1,8) |
| CD3^+^CD4^+^ Th (x10^9^/l) ^4^ | 1.00  (0,5-1,3) | 0.89  (0,5-1,3) | - | 0.85  (0,5-1,3) | 1.20  (0,5-1,8) | 0.70  (0,5-1,8) | 0.70  (0,5-1,8) | 0.66  (0,5-1,8) | - | 1.32  (0,5-1,8) | 0.70  (0,5-1,8) | - | - | - | - | - | 0.87  (0,7-2,0) | - | 1.26  (0,7-2,0) | - | 1.5  (0,7-2,0) | 1.57  (0,7-2,0) | - |
| CD19^+^ B (x10^9^/l) ^4^ | 0.20  (0,2-0,5) | 0.56  (0,2-0,5) | - | 0.63  (0,2-0,5) | 0.60  (0,3-0,7) | 0.30  (0,3-0,7) | 0.20  (0,3-0,7) | 0.23  (0,3-0,7) | - | 0.92  (0,3-0,7) | 0.70  (0,3-0,7) | - | - | - | - | - | 0.60  (0,4-1,5) | - | 0.44  (0,4-1,5) | - | 0.60  (0,4-1,5) | 0.51  (0,4-1,5) | - |
| B21lo (x10^9^/l) ^4^ | 0.0019  (0,0039-0,037) | 0.0008  (0,0039-0,037) | - | 0.0038  (0,0039-0,037) | 0.0073  (0,0059-0,036) | 0.0176  (0,0059-0,036) | 0.0200  (0,0059-0,036) | 0.0033  (0,0059-0,036) | - | 0.0026  (0,0059-0,036) | 0.0106  (0,0059-0,036) | - | - | - | - | - | 0.0029  (0,0069-0,099) | - | 0.0037  (0,0069-0,099) | - | - | 0.0126  (0,0069-0,099) | - |
| smB (x10^9^/l) ^4^ | 0.0014  (0,0065-0,073) | 0.0225  (0,0065-0,073) | - | 0.0152  (0,0065-0,073) | 0.0569  (0,0070-0,051) | 0.0149  (0,0070-0,051) | 0.0100  (0,0070-0,051) | 0.0226  (0,0070-0,051) | - | 0.0336  (0,0070-0,051) | 0.0365  (0,0070-0,051) | - | - | - | - | - | 0.0167  (0,0022-0,25) | - | 0.0134  (0,0022-0,25) | - | - | 0.0262  (0,0022-0,25) | - |
| granulocytes | nml nr | nml nr | nml nr | nml nr | nml nr | nml nr | nml nF | nml nr | nml nF | nml nF | nml nr | nml nr | nml nr | nml nr | nml nr | nml nr | nml nr | nml nr | nml nr | nml nr | nml nF | nml nr | nml nr |
| classical C pathway | nml | nml | nml | - | nml | nml | nml | - | nml | Nml | nml | nml | nml | - | nml | nml | nml | nml | nml | nml | - | nml | nml |
| alternative C pathway | nml | nml | nml | - | nml | nml | nml | - | nml | Nml | nml | nml | nml | - | nml | nml | nml | nml | nml | nml | - | nml | nml |
| MBL C pathway | - | nml | - | - | - | - | - | - | - | - | - | - | nml | - | nml | nml | snml | nml | - | - | - | nml | nml |
| ANA (titer) | >1:640 | neg | - | - | neg | 1:160 | neg | - | - | neg | - | - | neg | neg | - | neg | - | - | - | - | - | neg | - |
| Ferritin (nml: 25-250μg/l) | 15 | 35 | - | - | nml | nml | 18 | - | 48 | 13 | 48 | 9.4 | 9 | 50 | 16 | 11 | - | 78 | 14 | - | - | 44 | 21 |
| IgE (IU/ml) (nml: <50 ≤10yrs; <100 >10yrs) | 48 | 16 | 350 | - | 6 | 22 | 33 | 130 | 110 | 53 | 23 | 92 | 5 | 61 | 6 | 4 | 1100 | 24 | 24 | 40 | 410 | 460 | 19 |
| sIgE(s) | neg | neg | neg | neg | neg | neg | neg | neg | neg | pos | neg | neg | neg | neg | neg | - | pos | neg | neg | neg | neg | pos | neg |
| response diphtheria^5^ | 0.53 | 1.17 | - | 0.04 | 1.29 | 0.22 | 0.08 | - | 4.41 | 1.72 | 1.45 | 5.24 | - | - | 1.89 | 0.18 | 0.89 | - | 0.16 | 1.65 | - | 0.71 | 0.81 |
| response tetanus^5^ | >16 | >16 | - | 0.82 | 4.03 | 0.55 | 2.15 | - | 12.38 | 7.17 | 14.46 | 12.84 | - | - | 2.08 | 1.01 | 13.19 | - | 6.97 | 2.04 | - | 1.56 | 1.17 |
| response Pneumo23^6^ | abs | nml | - | snml | snml | snml | snml | - | nml | snml | nml | nml | nml | - | nml | nml | nml | - | nml | nml | - | nml | snml |
| QoL-parents body^7^ | 28 | - | 26 | 26 | 26 | 27 | 28 | 28 | 28 | 27 | 26 | 30 | 28 | - | - | 27 | - | 29 | - | - | - | - | - |
| QoL-child body^7^ | 26 | - | 18 | 17 | 12 | 28 | 23 | 25 | - | - | - | - | - | - | - | - | - | - | - | - | - | - | - |
| *reference p10-p90*^7^ | 16-30 | - | 16-30 | 16-30 | 18-31 | 18-31 | 18-31 | 18-31 | - | - | - | - | - | - | - | - | - | 16-30 | - | - | - | - | - |
| QoL-parents motor^7^ | 28 | - | 31 | 32 | 30 | 30 | 32 | 27 | 30 | 29 | 31 | 32 | 32 | - | - | 24 | - | 32 | - | - | - | - | - |
| QoL-child motor^7^ | 24 | - | 32 | 30 | 15 | 27 | 30 | 25 | - | - | - | - | - | - | - | - | - | - | - | - | - | - | - |
| *reference p10-p90*^7^ | 26-32 | - | 26-32 | 26-32 | 26-32 | 26-32 | 26-32 | 26-32 | - | - | - | - | - | - | - | - | - | 26-32 | - | - | - | - | - |
| QoL-parents cogn^7^ | 25 | - | 28 | 27 | 30 | 28 | 32 | 25 | 28 | 31 | 32 | 30 | 31 | - | - | 32 | - | 30 | - | - | - | - | - |
| QoL-child cogn^7^ | 26 | - | 23 | 30 | 25 | 27 | 32 | 31 | - | - | - | - | - | - | - | - | - | - | - | - | - | - | - |
| *reference p10-p90*^7^ | 22-32 | - | 22-32 | 22-32 | 23-32 | 23-32 | 23-32 | 23-32 | - | - | - | - | - | - | - | - | - | 22-32 | - | - | - | - | - |
| QoL-parents social^7^ | 32 | - | 32 | 32 | 32 | 32 | 30 | 32 | 32 | 30 | 30 | 32 | 32 | - | - | 28 | - | 32 | - | - | - | - | - |
| QoL-child social^7^ | 32 | - | 32 | 32 | 32 | 32 | 30 | 32 | - | - | - | - | - | - | - | - | - | - | - | - | - | - | - |
| *reference p10-p90*^7^ | 29-32 | - | 29-32 | 29-32 | 26-32 | 26-32 | 26-32 | 26-32 | - | - | - | - | - | - | - | - | - | 29-32 | - | - | - | - | - |
| QoL-parents pos^7^ | 12 | - | 16 | 16 | 9 | 15 | 15 | 14 | 16 | 14 | 14 | 16 | 15 | - | - | 15 | - | 16 | - | - | - | - | - |
| QoL-child pos^7^ | 13 | - | 16 | 16 | 9 | 16 | 15 | 15 | - | - | - | - | - | - | - | - | - | - | - | - | - | - | - |
| *reference p10-p90*^7^ | 9-16 | - | 9-16 | 9-16 | 10-16 | 10-16 | 10-16 | 10-16 | - | - | - | - | - | - | - | - | - | 9-16 | - | - | - | - | - |
| QoL-parents neg^7^ | 12 | - | 14 | 15 | 4 | 12 | 11 | 16 | 14 | 9 | 9 | 14 | 11 | - | - | 10 | - | 10 | - | - | - | - | - |
| QoL-child neg^7^ | 13 | - | 14 | 16 | 4 | 13 | 11 | 13 | - | - | - | - | - | - | - | - | - | - | - | - | - | - | - |
| *reference p10-p90*^7^ | 8-15 | - | 8-15 | 8-15 | 8-15 | 8-15 | 8-15 | 8-15 | - | - | - | - | - | - | - | - | - | 8-15 | - | - | - | - | - |
| QoL stomach^8^ | - | - | - | - | - | - | - | - | - | - | - | - | - | 50 | 100 | - | 92 | - | 67 | 100 | 33 | 50 | 67 |
| *reference p10-p90*^8^ | - | - | - | - | - | - | - | - | - | - | - | - | - | 73-100 | 73-100 | - | 73-100 | - | 73-100 | 73-100 | 73-100 | 73-100 | 73-100 |
| QoL skin^8^ | - | - | - | - | - | - | - | - | - | - | - | - | - | 75 | 100 | - | 75 | - | 92 | 100 | 100 | 100 | 33 |
| *reference p10-p90*^8^ | - | - | - | - | - | - | - | - | - | - | - | - | - | 75-100 | 75-100 | - | 75-100 | - | 75-100 | 75-100 | 75-100 | 75-100 | 75-100 |
| QoL lung^8^ | - | - | - | - | - | - | - | - | - | - | - | - | - | 100 | 67 | - | 50 | - | 100 | 100 | 50 | 67 | 67 |
| *reference p10-p90*^8^ | - | - | - | - | - | - | - | - | - | - | - | - | - | 75-100 | 75-100 | - | 75-100 | - | 75-100 | 75-100 | 75-100 | 75-100 | 75-100 |
| QoL sleeping^8^ | - | - | - | - | - | - | - | - | - | - | - | - | - | 100 | 100 | - | 100 | - | 94 | 88 | 38 | 88 | 81 |
| *reference p10-p90*^8^ | - | - | - | - | - | - | - | - | - | - | - | - | - | 56-100 | 56-100 | - | 56-100 | - | 56-100 | 56-100 | 56-100 | 56-100 | 56-100 |
| QoL appetite^8^ | - | - | - | - | - | - | - | - | - | - | - | - | - | 92 | 67 | - | 100 | - | 67 | 75 | 33 | 92 | 58 |
| *reference p10-p90*^8^ | - | - | - | - | - | - | - | - | - | - | - | - | - | 75-100 | 75-100 | - | 75-100 | - | 75-100 | 75-100 | 75-100 | 75-100 | 75-100 |
| QoL lively^8^ | - | - | - | - | - | - | - | - | - | - | - | - | - | 50 | 67 | - | 100 | - | 100 | 100 | 67 | 100 | 67 |
| *reference p10-p90*^8^ | - | - | - | - | - | - | - | - | - | - | - | - | - | 100-100 | 100-100 | - | 100-100 | - | 100-100 | 100-100 | 100-100 | 100-100 | 100-100 |
| QoL pos^8^ | - | - | - | - | - | - | - | - | - | - | - | - | - | 100 | 100 | - | 100 | - | 100 | 100 | 83 | 100 | 100 |
| *reference p10-p90*^8^ | - | - | - | - | - | - | - | - | - | - | - | - | - | 100-100 | 100-100 | - | 100-100 | - | 100-100 | 100-100 | 100-100 | 100-100 | 100-100 |
| QoL probl behav^8^ | - | - | - | - | - | - | - | - | - | - | - | - | - | 57 | 79 | - | 93 | - | 71 | 71 | 50 | 50 | 57 |
| *reference p10-p90*^8^ | - | - | - | - | - | - | - | - | - | - | - | - | - | 50-86 | 50-86 | - | 50-86 | - | 50-86 | 50-86 | 50-86 | 50-86 | 50-86 |
| QoL anxiety^8^ | - | - | - | - | - | - | - | - | - | - | - | - | - | 50 | 50 | - | 67 | - | 67 | 83 | 100 | 67 | 67 |
| *reference p10-p90*^8^ | - | - | - | - | - | - | - | - | - | - | - | - | - | 50-100 | 50-100 | - | 50-100 | - | 50-100 | 50-100 | 50-100 | 50-100 | 50-100 |
| QoL social^8^ | - | - | - | - | - | - | - | - | - | - | - | - | - | 100 | 100 | - | 100 | - | 100 | 100 | 100 | 100 | 100 |
| *reference p10-p90*^8^ | - | - | - | - | - | - | - | - | - | - | - | - | - | 67-100 | 67-100 | - | 67-100 | - | 67-100 | 67-100 | 67-100 | 67-100 | 67-100 |
| QoL motor^8^ | - | - | - | - | - | - | - | - | - | - | - | - | - | 100 | 88 | - | 100 | - | 100 | 100 | 100 | 100 | 100 |
| *reference p10-p90*^8^ | - | - | - | - | - | - | - | - | - | - | - | - | - | 94-100 | 94-100 | - | 94-100 | - | 94-100 | 94-100 | 94-100 | 94-100 | 94-100 |
| QoL communication^8^ | - | - | - | - | - | - | - | - | - | - | - | - | - | 81 | 88 | - | 100 | - | 81 | 100 | 81 | 100 | 88 |
| *reference p10-p90*^8^ | - | - | - | - | - | - | - | - | - | - | - | - | - | 81-100 | 81-100 | - | 81-100 | - | 81-100 | 81-100 | 81-100 | 81-100 | 81-100 |

^1^ According to the eight clinical presentations of primary immunodeficiency described in reference (1). ^2^ According to the working diagnoses used in the ESID

online Registry described in reference (2). For sIgA and sIgM, the absence of *clinical* signs of T-cell deficiency was considered sufficient. When taking all details

of the ESID Registry Working Diagnoses into account, sIgA, sIgM and uAD in these patients all fall under the Working Diagnosis ‘unclassified antibody

deficiency’ (unPAD). ^3^ Prior to or no immunoglobulin substitution. ^4^ Age-related reference values in brackets, according to reference (1). ^5^ Response to

vaccination with diphtheria-tetanus-polyomyelitis booster vaccination was determined (pre-post vaccination titers in IU/ml). ^6^ Response to vaccination with

Pneumo23 polysaccharide 23-valent pneumococcal vaccination (normal[nml], subnormal[snml], absent[abs]; according to the reference values of the laboratory

doing the tests, only serotypes not influenced by protein-conjugated vaccination received previously are used for the evaluation of the results). ^7^ Determined

using the TNO Quality of Life Questionnaires for children TAC-QOL parent resp. child questionnaire, 10^th^-90^th^ centile values of the Dutch age-related reference

population (8-11 and 12-15yrs resp.)(3); body = problems /limitations concerning general physical functioning/complaints; motor = problems / limitations

concerning motor functioning; auto = problems / limitations concerning independent daily functioning; cogn = problems / limitations concerning cognitive

functioning and school performances; social = problems / limitations in social contacts, with parents and peers; pos = the occurrence of positive moods; neg =

the occurrence of negative moods. ^8^ Determined using the TNO Quality of Life Questionnaires for children TAP-QOL questionnaire, 10^th^-90^th^ centile values of

the Dutch age-related reference population(4); stomach = measures stomach and intestinal problems; skin = measures skin problems like eczema, itchiness,

and dry skin; lung = measures difficulties with breathing, lung problems, bronchitis or shortness of breath; sleeping = measures sleeping problems like being

awake or crying or difficulty sleeping during the night; appetite = measures if the child had a bad appetite, difficulty to eat enough of refused to eat; probl

behav = measures difficult and aggressive behavior of the child; pos = measures positive emotions; anxiety = measures if the child was anxious, tense or

frightened; lively = measures if the child was active, lively and energetic; social = measures social contacts with other children, like if the child was at ease

with other children; motor = measures gross motor problems like difficulties with walking, running, climbing stairs and balance; communication = measures

communicative skills of the child when compared to children of the same age. Abbreviations: red = value outside age-related reference; - = not available, not

performed; ab = daily antibiotic prophylaxis; ail = the clinical presentation ‘autoimmune or chronic inflammatory disease; lymphoproliferation’; ANA =

antinuclear antibody; B = B-lymphocytes; B21lo = CD19^+^CD38^low^CD21^low^ B-lymphocytes; BHR = bronchial hyperreactivity; brp = bronchopathy (according to

the attending radiologist); C = complement; C1 = child with study number 1; CD = cluster of differentiation; cITP = chronic immune thrombocytopenic

purpura; eci = ‘e causa ignota’ (cause not found); ENT = ear-nose-throat; F = female; fe = 3 months of oral iron supplementation; ftt = the clinical presentation

‘failure to thrive from early infancy’; g/l = gram per liter; HRCT = high resolution computerized tomography; Ig = immunoglobulin; iron = deficient stores (low

ferritin); IU/ml = international units per milliliter; iv = intravenous immunoglobulin substitution; lo = low (surface expression); lym = lymphocytes; M = male;

MBL = mannose binding lectin deficiency; ml = milliliter; N = no; neg = negative; nf = number + function; nml = normal; nr = number; PAD = primary antibody

deficiency; pos = positive; pyo = the clinical presentation ‘recurrent pyogenic infections’; res = the clinical presentation ‘recurrent ENT and airway infections’;

sc = subcutaneous immunoglobulin substitution; sIgA = selective IgA deficiency (absence of *clinical* signs of T-cell deficiency was considered sufficient); sIgE(s)

= positive specific IgE antibody/antibodies in serum; sIgM = selective IgM deficiency (absence of *clinical* signs of T-cell deficiency was considered sufficient);

smB = switched memory B-lymphocytes (CD19^+^CD27^+^IgD^-^IgM^-^ lymphocytes); snml = subnormal (but not absent); Th = T-helper lymphocytes; THI = the clinical

presentation ‘transient hypogammaglobulinaemia of infancy’ (the immunological abnormalities resolved during the study); uAD = unclassified antibody

deficiency; un = the clinical presentation ‘unusual infections or unusually severe course of infections’; Y = yes; yrs = years.

**References**

1. de Vries E. Patient-centred screening for primary immunodeficiency, a multi-stage diagnostic protocol designed for non-immunologists: 2011 update. Clin Exp Immunol. 2012 Jan;167(1):108–19.

2. Edgar D, Ehl S. ESID Registry - Working definitions for clinical diagnosis of PID. 2016;1–9.

3. Nederlandse D. Vragenlijsten kwaliteit van leven.

4. TACQOL CF 8-11 Reference data. https://www.tno.nl/nl/aandachtsgebieden/gezond-leven/prevention-work-health/gezond-en-veilig-opgroeien/vragenlijsten-gezondheidsrelateerde-kwaliteit-van-leven/
