# Supplementary material for: Mild Hypogammaglobulinemia Can Be a Serious Condition
Source: Front Immunol. 2018 Oct 15;9:2384. doi: 10.3389/fimmu.2018.02384 (PMC6196282; doi:10.3389/fimmu.2018.02384)
Supplement: Supplementary Table 2 — Descriptive statistics in 99 adults with primary antibody deficiency. [file Table_2.docx]

**Supplementary table 2. Descriptive statistics in 99 adults with primary antibody deficiency.**

| **Continuous variables** | | | | | | | | | | | | | | | | | | | | | | | | | |
| --- | --- | --- | --- | --- | --- | --- | --- | --- | --- | --- | --- | --- | --- | --- | --- | --- | --- | --- | --- | --- | --- | --- | --- | --- | --- |
| *Variable* | | | | *Number of responses* | | | | | | | | | | | *Mean* | | | | | | | *Standard deviation* | | | |
| BMI | | | | 95 | | | | | | | | | | | 26.4 | | | | | | | 5.1 | | | |
| Smoking pack years | | | | 97 | | | | | | | | | | | 7 | | | | | | | 0.20 | | | |
| **Categorical variables** | | | | | | | | | | | | | | | | | | | | | | | | | |
| *Variable* | | | | *Category* | | | | | | | | | | | *Number* | | | | | | | *Percentage* | | | |
| Referred by | | | | Allergologist  ENT  Gastroenterologist  General Practitioner  Internist  Paediatrician  Pulmonologist | | | | | | | | | | | 1  2  1  35  15  2  43 | | | | | | | 1%  2%  1%  35%  15%  2%  43% | | | |
| Diagnosis | | | | CVID  Selective IgA deficiency  Selective IgM deficiency  Unclassified antibody deficiency | | | | | | | | | | | 10  4  4  81 | | | | | | | 10%  4%  4%  82% | | | |
| HRCT bronchial wall thickening | | | | No  Yes | | | | | | | | | | | 31  29 | | | | | | | 52%  48% | | | |
| HRCT bronchiectasis | | | | None  1 lobe  2 / 3 lobes  4+ lobes | | | | | | | | | | | 31  9  10  10 | | | | | | | 52%  15%  17%  17% | | | |
| HRCT mucus plugging | | | | None  Central  Peripheral | | | | | | | | | | | 49  3  8 | | | | | | | 82%  5%  13% | | | |
| Atelectasis | | | | No  Yes | | | | | | | | | | | 50  10 | | | | | | | 83%  17% | | | |
| First clinical presentation | | | | Autoimmunity, chronic inflammation  ENT-airway infections  Unusual infections | | | | | | | | | | | 10  88  1 | | | | | | | 10%  89%  1% | | | |
| Prescribed therapy (related to antibody deficiency diagnosis) | | | | None  Prophylactic antibiotics  IVIG  SCIG | | | | | | | | | | | 50  20  23  6 | | | | | | | 51%  20%  23%  6% | | | |
| Familial case | | | | No  Yes | | | | | | | | | | | 79  20 | | | | | | | 80%  20% | | | |
| Gender | | | | Male  Female | | | | | | | | | | | 28  71 | | | | | | | 28%  72% | | | |
| Highest education level | | | | None  VMBO (pre-MBO)  HAVO (pre-HBO)  MBO (practical education)  HBO (applied science)  WO (university) | | | | | | | | | | | 21  17  5  31  13  9 | | | | | | | 22%  18%  5%  32%  14%  9% | | | |
| **Summaries of patient’s family history** | | | | | | | | | | | | | | | | | | | | | | | | | |
| *Symptom* | | | | | | | | | *Number of responses* | | | | | | | | | | *Number* | | | | | *Percentage* | |
| Asthma  Chronic bronchitis  COPD  Food allergy  Allergy to inhaled material  Allergy to animals  Being ‘always ill’  Eczema  Chronic sinusitis  Chronic otitis  Grommet placement  Adenotonsillectomy  Young people dying  Deafness  Autoimmune disease  Rheumatoid arthritis  Thyroid problems  Coeliac disease  Crohn’s disease  Ulcerative colitis  Chronic intestinal inflammation  Hay fever  Cancer  Leukemia  Lymphoma  Being ‘always tired’ | | | | | | | | | 98  98  98  98  98  98  97  98  98  98  98  98  98  98  98  98  98  98  98  98  98  98  98  98  98  98 | | | | | | | | | | 53  46  32  26  39  29  28  43  30  12  40  67  38  38  25  48  33  10  8  7  14  46  68  11  4  35 | | | | | 54%  47%  33%  27%  40%  30%  29%  44%  31%  12%  41%  68%  39%  39%  26%  49%  34%  10%  8%  7%  14%  47%  69%  11%  4%  36% | |
| **Combined family history*** | | | | | | | | | | | | | | | | | | | | | | | | | |
| *Symptoms* | | | | | | | | | *Number of responses* | | | | | | | | | | *Number* | | | | | *Percentage* | |
| Pulmonary (COPD, asthma, bronchitis)  Autoimmune (autoimmunity, rheumatoid arthritis, thyroid problems)  IBD (Crohn’s disease, ulcerative colitis, intestinal inflammation)  Ear (sinusitis, otitis)  Fatigue (always ill, always tired)  Cancer (leukemia, lymphoma)  Allergy (allergy to food, material, animals, eczema, hay fever)  Pulmonary and/or ear  Cancer, IBD and/or autoimmune | | | | | | | | | 98  98  98  98  98  98  97  98  98 | | | | | | | | | | 71  66  20  37  43  13  70  78  73 | | | | | 72%  67%  20%  38%  44%  13%  71%  80%  74% | |
| **Disease status** | | | | | | | | | | | | | | | | | | | | | | | | | |
| *Variable* | | | | | | *Category* | | | | | | | | | | | *Number* | | | | | | *Percentage* | | |
| Patient always ill | | | | | | Yes  No (same as other people)  No (less frequently than others) | | | | | | | | | | | 87  8  3 | | | | | | 89%  8%  3% | | |
| Most important complaint | | | | | | Airway infection  Chronic cough  ENT infection  Tired, no energy  Other | | | | | | | | | | | 35  4  9  35  15 | | | | | | 36%  4%  9%  36%  15% | | |
| **Summaries of previous symptoms** (yes/no binary variables) | | | | | | | | | | | | | | | | | | | | | | | | | |
| *Symptom* | | | | | | *Number of responses* | | | | | | | | | | | *Number* | | | | | | *Percentage* | | |
| Problems with vision  Problems with hearing  Otitis in the past  Sinusitis in the past  Grommets placed (past)  Adenotonsillectomy (past)  Otitis nowadays  Sinusitis nowadays  Surgery due to chronic sinusitis  Dyspnoea (at rest)  Dyspnoea (exercising)  Dyspnoea (change in temperature)  Dyspnoea (cigarette smoke)  Dyspnoea (strong smells)  Coughing productive  Bladder infections  Watery diarrhea  Diarrhea (not watery)  Eczema  Other skin problems  Pain in your bones/joints  Red/swollen joints  Chest pain during exercise | | | | | | 98  98  98  98  98  98  97  98  98  98  98  98  98  98  98  98  98  98  98  98  98  98  98 | | | | | | | | | | | 38  30  33  51  10  53  10  41  17  35  61  47  52  42  41  29  1  12  25  42  70  24  19 | | | | | | 39%  31%  34%  52%  10%  54%  10%  42%  17%  36%  62%  48%  53%  43%  42%  30%  1%  12%  26%  43%  71%  24%  19% | | |
| **Summaries of previous symptoms** (variables with multiple categories) | | | | | | | | | | | | | | | | | | | | | | | | | |
| Suffer coughing | | | Never  Sometimes  Daily <8 weeks  Chronic >8 weeks | | | | | | | 3  51  17  27 | | | | | | | | 3%  52%  17%  28% | | | | | | | |
| Season of most complaints | | | Spring  Summer  Autumn  Winter | | | | | | | 16  14  14  52 | | | | | | | | 17%  15%  15%  54% | | | | | | | |
| How is your appetite | | | Bad  All Right  Good | | | | | | | 5  30  63 | | | | | | | | 5%  31%  64% | | | | | | | |
| **Summaries of TAAQOOL domain scores** | | | | | | | | | | | | | | | | | | | | | | | | | |
| *TAAQOL domain* | | | *Number of responses* | | | | | *Median* | | | | | | | | | | *Inter-quartile range* | | | | | | | |
| Gross motor functioning  Fine Motor functioning  Cognition  Sleep  Pain  Social contacts  Daily activities  Sex  Vitality  Happiness  Depressive mood  Anger | | | 98  98  98  98  98  98  97  97  98  98  98  97 | | | | | 63  100  63  56  50  81  50  88  29  50  75  89 | | | | | | | | | | 25, 88  75, 100  31, 100  31, 81  36, 75  63, 100  22, 88  50, 100  8, 50  33, 67  50, 83  67, 100 | | | | | | | |
| **Summaries of original TAAQOL questions (Q1-30, part 1)** | | | | | | | | | | | | | | | | | | | | | | | | | |
| *Question* | | | | | | | | *No* | | *A little* | | | | | | | | *Some* | | | | | | | *A lot* |
| 1. Difficulty walking up stairs  2. Difficulty bending over  3. Difficulty walking 500 yards  4. Difficulty lifting  5. Difficulty with scissors  6. Difficulty fastening buttons  7. Difficulty opening a can  8. Difficulty twisting jar lid  9. Difficulty concentrating on others  10. Difficulty remembering things  11. Difficulty concentrated thinking  12. Mind wandered | | | | | | | | 37 (38%)  46 (47%)  48 (49%)  41 (42%)  83 (84%)  77 (79%)  71 (72%)  63 (64%)  51 (52%)  40 (41%)  42 (43%)  40 (41%) | | 24 (24%)  20 (20%)  18 (18%)  23 (23%)  6 (6%)  13 (13%)  11 (11%)  14 (14%)  23 (23%)  28 (14%)  26 (27%)  26 (27%) | | | | | | | | 17 (17%)  18 (18%)  21 (21%)  19 (19%)  3 (13%)  2 (2%)  11 (11%)  14 (14%)  11 (11%)  14 (14%)  12 (12%)  11 (11%) | | | | | | | 20 (20%)  14 (14%)  11 (11%)  15 (15%)  6 (6%)  6 (6%)  5 (5%)  7 (7%)  13 (13%)  16 (16%)  18 (18%)  21 (21%) |
| *Question* | | | | | | | | *Never* | | *Occasionally* | | | | | | | | *Often* | | | | | | | *Always* |
| 13. Difficulty getting to sleep  14. Slept restlessly  15. Lay awake a lot at night  16. Good night’s sleep  17. Back-ache  18. Pain in neck/shoulders  19. Pain in joints/limbs  20. Pain in muscles | | | | | | | | 32 (33%)  26 (27%)  34 (35%)  11 (11%)  35 (36%)  24 (24%)  29 (30%)  33 (34%) | | 46 (47%)  38 (39%)  38 (39%)  31 (32%)  31 (32%)  28 (29%)  27 (28%)  30 (31%) | | | | | | | | 10 (10%)  23 (23%)  17 (17%)  25 (26%)  17 (17%)  28 (29%)  29 (30%)  21 (21%) | | | | | | | 10 (10%)  11 (11%)  9 (9%)  31 (32%)  15 (15%)  18 (18%)  13 (13%)  14 (14%) |
| *Question* | | | | | | | | *Often* | | *Occasionally* | | | | | | | | *Seldom* | | | | | | | *Never* |
| 21. Talk others in confidence  22. Nice time with other people  23. Visit friends  24. Have good time with others | | | | | | | | 63 (64%)  55 (56%)  44 (45%)  54 (55%) | | 23 (23%)  26 (27%)  33 (34%)  30 (31%) | | | | | | | | 6 (6%)  11 (11%)  15 (15%)  9 (9%) | | | | | | | 6 (6%)  6 (6%)  6 (6%)  5 (5%) |
| *Question* | | | | | | | | *No* | | | | | | *A little* | | | | *Some* | | | | | | | *A lot* |
| 25. Difficulty with work/study  26. Done less work/study  27. Problems with types work  28. Worked less conscientiously  29. Less sex  30. Found sex less satisfying | | | | | | | | 30 (31%)  39 (41%)  31 (32%)  47 (48%)  49 (51%)  64 (66%) | | | | | | 17 (18%)  18 (19%)  21 (22%)  19 (20%)  14 (14%)  11 (11%) | | | | 28 (29%)  18 (19%)  26 (27%)  19 (20%)  16 (16%)  13 (13%) | | | | | | | 22 (23%)  21 (22%)  19 (20%)  12 (12%)  18 (19%)  9 (9%) |
| **Summaries of original TAAQOL questions (Q1-30, part 2)** | | | | | | | | | | | | | | | | | | | | | | | | | |
| *Question* | | | | | | | | *Not at all* | | | | *A little* | | | | | | *Quite a lot* | | | | | | | *Very much* |
| 1. Difficulty walking up stairs  2. Difficulty bending over  3. Difficulty walking 500 yards  4. Difficulty lifting  5. Difficulty with scissors  6. Difficulty fastening buttons  7. Difficulty opening a can  8. Difficulty twisting jar lid  9. Difficulty concentrating on others  10. Difficulty remembering things  11. Difficulty concentrated thinking  12. Mind wandered  13. Difficulty getting to sleep  14. Slept restlessly  15. Lay awake a lot at night  16. Good night’s sleep  17. Back-ache  18. Pain in neck/shoulders  19. Pain in joints/limbs  20. Pain in muscles  21. Talk others in confidence  22. Nice time with other people  23. Visit friends  24. Have good time with others  25. Difficulty with work/study  26. Done less work/study  27. Problems with types work  28. Worked less conscientiously  29. Less sex  30. Found sex less satisfying | | | | | | | | 3 (5%)  3 (6%)  3 (6%)  4 (7%)  3 (20%)  3 (14%)  3 (11%)  3 (9%)  4 (9%)  5 (9%)  3 (5%)  11 (19%)  11 (17%)  6 (8%)  5 (8%)  23 (35%)  4 (6%)  5 (7%)  4 (6%)  9 (14%)  14 (41%)  7 (17%)  16 (30%)  19 (44%)  1 (1%)  2 (4%)  0 (0%)  4 (8%)  10 (21%)  7 (21%) | | | | 20 (33%)  15 (29%)  14 (28%)  19 (33%)  6 (40%)  11 (52%)  9 (33%)  13 (37%)  14 (30%)  15 (26%)  20 (36%)  17 (29%)  32 (48%)  36 (50%)  28 (44%)  18 (27%)  25 (40%)  29 (39%)  25 (36%)  26 (40%)  15 (44%)  12 (29%)  13 (25%)  10 (24%)  14 (21%)  17 (30%)  22 (33%)  16 (32%)  20 (42%)  11 (33%) | | | | | | 19 (31%)  17 (33%)  8 (16%)  15 (26%)  4 (27%)  5 (24%)  9 (33%)  11 (31%)  18 (38%)  22 (38%)  15 (27%)  20 (34%)  11 (17%)  18 (25%)  18 (28%)  13 (20%)  15 (24%)  21 (28%)  27 (39%)  19 (29%)  3 (9%)  10 (24%)  16 (30%)  12 (28%)  23 (34%)  19 (33%)  17 (26%)  12 (24%)  12 (25%)  8 (24%) | | | | | | | 19 (31%)  17 (33%)  25 (50%)  19 (33%)  2 (13%)  2 (10%)  6 (22%)  8 (23%)  11 (23%)  16 (28%)  18 (32%)  10 (17%)  12 (18%)  12 (17%)  13 (20%)  12 (18%)  19 (30%)  19 (26%)  13 (19%)  11 (17%)  2 (6%)  13 (31%)  8 (15%)  2 (5%)  29 (43%)  19 (33%)  27 (41%)  18 (36%)  6 (13%)  7 (21%) |
| **Summaries of original TAAQOL questions (Q31-45)** | | | | | | | | | | | | | | | | | | | | | | | | | |
| *Question* | | | | | | | | *No* | | *A little* | | | | | | | | *Quite* | | | | | | | *Very* |
| 31. Energetic  32. Tired  33. Fit  34. Exhausted quickly  35. Joyful  36. Sad  37. In good spirits  38. Angry  39. Worried  40. Gloomy  41. Aggressive  42. Happy  43. Short-tempered  44. Cheerful  45. Anxious | | | | | | | | 45 (46%)  10 (10%)  48 (49%)  15 (15%)  13 (13%)  35 (36%)  19 (20%)  44 (45%)  19 (19%)  44 (45%)  71 (72%)  6 (6%)  69 (71%)  11 (11%)  49 (50%) | | 28 (29%)  20 (20%)  29 (30%)  19 (19%)  36 (37%)  41 (42%)  30 (13%)  34 (35%)  29 (30%)  35 (36%)  19 (19%)  31 (32%)  22 (23%)  32 (33%)  28 (29%) | | | | | | | | 20 (20%)  34 (35%)  16 (16%)  30 (31%)  35 (36%)  17 (17%)  37 (38%)  11 (11%)  31 (32%)  10 (10%)  5 (5%)  40 (41%)  4 (4%)  46 (47%)  13 (13%) | | | | | | | 5 (5%)  34 (35%)  5 (5%)  34 (35%)  14 (14%)  5 (5%)  11 (11%)  9 (9%)  19 (19%)  9 (9%)  3 (3%)  21 (21%)  2 (2%)  9 (9%)  8 (8%) |
| **Summaries of normally distributed laboratory test results** | | | | | | | | | | | | | | | | | | | | | | | | | |
| *Variable* | | | | | | | *Number of responses* | | | | | | | | | *Mean* | | | | *Standard deviation* | | | | | |
| Leukocyte count (10^9^/l)  Thrombocyte count (10^9^/l)  Lymphocyte count (10^9^/l)  Monocyte count (10^9^/l)  T-helper lymphocytes (10^9^/l) | | | | | | | 98  98  98  98  84 | | | | | | | | | 7.6  270  2.1  0.42  0.94 | | | | 2.8  73  0.7  0.14  0.40 | | | | | |
| Classical complement pathway activity (%)  Alternative complement pathway activity (%) | | | | | | | 80  80 | | | | | | | | | 109  100 | | | | 24  25 | | | | | |
| **Summaries of non-normally distributed laboratory test results** | | | | | | | | | | | | | | | | | | | | | | | | | |
| *Variable* | | | | | | | *Number of responses* | | | | | | | | *Median* | | | | | *Inter-quartile range* | | | | | |
| Granulocyte count (10^9^/l)  Eosinophil count (10^9^/l)  Basophil count (10^9^/l)  Erythrocyte sedimentation rate (mm/1^st^ hour)  CRP (mg/l)  T lymphocytes (10^9^/l)  Cytotoxic T lymphocytes (10^9^/L)  Natural killer cells (10^9^/l)  B lymphocytes (10^9^/l)  Total memory B lymphocytes (10^9^/l)  Non-switched memory B lymphocytes (10^9^/l)  Switched memory B lymphocytes (10^9^/l)  CD21 low B lymphocytes (10^9^/l)  Naive mature B lymphocytes (10^9^/l)  Transitional B lymphocytes (10^9^/l)  Plasmablasts (10^9^/l) | | | | | | | 98  98  98  74  71  84  84  83  84  74  74  74  74  74  74  74 | | | | | | | | 4.2  0.1  0.0  4  3  1.40  0.48  0.22  0.20  0.05  0.003  0.019  0.006  0.09  0.008  0.0013 | | | | | 3.2, 5.9  0.1, 0.2  0.0, 0.1  2, 12  3, 6  1.12, 1.90  0.31, 0.68  0.15, 0.34  0.11, 0.30  0.03, 0.08  0.001, 0.007  0.009, 0.036  0.003, 0.010  0.05, 0.16  0.003, 0.015  0.0005, 0.0037 | | | | | |
| Total IgG (g/l)  IgA (g/l)  IgM (g/l)  IgG1 subclass (g/l)  IgG2 subclass (g/l)  IgG3 subclass (g/l)  IgG4 subclass (g/l)  Total IgE (IU/ml) | | | | | | | 99  99  99  96  96  96  96  86 | | | | | | | | 7.6  1.4  0.6  5.1  1.5  0.31  0.17  23 | | | | | 6.1, 9.6  0.7, 2.3  0.4, 1.0  4.0, 6.2  1.2, 2.3  0.21, 0.43  0.07, 0.39  7, 72 | | | | | |
| Diphtheria titer before vaccination (IU/ml)  Diphtheria titer after vaccination (IU/ml)  Tetanus titer before vaccination (IU/ml)  Tetanus titer after vaccination (IU/ml) | | | | | | | 86  80  86  80 | | | | | | | | 0.05  0.57  0.74  8.7 | | | | | 0.02, 0.12  0.24, 1.43  0.17, 1.49  2.8, 16.0 | | | | | |
| **Summaries of values below, within or above age-related reference ranges** | | | | | | | | | | | | | | | | | | | | | | | | | |
| *Parameter* | *Normal range* | | | | *Below* | | | | | | *In Range* | | | | | | | | | | *Above* | | | | |
| Lymphocyte (10^9^/l) | 1.1 - 2.5 | | | | 8 (8%) | | | | | | 70 (71%) | | | | | | | | | | 20 (20%) | | | | |
| IgG (g/l)  IgA (g/l)  IgM (g/l) | 7.0 - 16.0  0.7 - 4.0  0.4 - 2.3 | | | | 41 (41%)  24 (24%)  33 (33%) | | | | | | 53 (54%)  69 (70%)  64 (65%) | | | | | | | | | | 5 (5%)  6 (6%)  2 (2%) | | | | |
| IgG1 (g/l)  IgG2 (g/l)  IgG3 (g/l)  IgG4 (g/l) | 4.9 – 11.4  1.5 – 6.4  0.2 – 1.1  0.08 – 1.4 | | | | 44 (46%)  51 (53%)  20 (21%)  27 (28%) | | | | | | 50 (52%)  44 (46%)  74 (77%)  68 (71%) | | | | | | | | | | 2 (2%)  1 (1%)  2 (2%)  1 (1%) | | | | |
| **Summaries of pneumococcal serotypes** | | | | | | | | | | | | | | | | | | | | | | | | | |
| *Before vaccination* | | | *Number of responses* | | | | | *Median* | | | | | | | | | | *Inter-quartile range* | | | | | | | |
| Serotype 1 (microg/ml)  Serotype 3 (microg/ml)  Serotype 4 (microg/ml)  Serotype 5 (microg/ml)  Serotype 6 (microg/ml)  Serotype 7f (microg/ml)  Serotype 8 (microg/ml)  Serotype 9v (microg/ml)  Serotype 14 (microg/ml)  Serotype 15b (microg/ml)  Serotype 18c (microg/ml)  Serotype 19f (microg/ml)  Serotype 20 (microg/ml)  Serotype 23f (microg/ml)  Serotype 33f (microg/ml) | | | 43  43  43  42  88  43  45  89  88  45  44  89  45  88  45 | | | | | 0.24  0.26  0.12  0.13  0.13  0.33  0.21  0.11  0.40  0.12  0.52  0.27  0.33  0.15  0.28 | | | | | | | | | | 0.13, 0.68  0.08, 0.60  0.04, 0.27  0.04, 0.47  0.04, 0.37  0.08, 0.78  0.12, 0.41  0.04, 0.27  0.10, 1.95  0.00, 0.39  0.11, 0.91  0.08, 1.75  0.13, 1.25  0.04, 0.80  0.08, 0.66 | | | | | | | |
| *After vaccination* | | | *Number of responses* | | | | | *Median* | | | | | | | | | | *Inter-quartile range* | | | | | | | |
| Serotype 1 (microg/ml)  Serotype 3 (microg/ml)  Serotype 4 (microg/ml)  Serotype 5 (microg/ml)  Serotype 6 (microg/ml)  Serotype 7f (microg/ml)  Serotype 8 (microg/ml)  Serotype 9v (microg/ml)  Serotype 14 (microg/ml)  Serotype 15b (microg/ml)  Serotype 18c (microg/ml)  Serotype 19f (microg/ml)  Serotype 20 (microg/ml)  Serotype 23f (microg/ml)  Serotype 33f (microg/ml) | | | 44  43  43  43  85  44  42  86  86  43  43  86  43  86  43 | | | | | 1.4  0.9  0.4  1.7  0.7  1.5  2.9  0.6  3.3  1.8  1.9  0.9  1.1  0.6  1.6 | | | | | | | | | | 0.3, 4.3  0.4, 3.8  0.1, 1.5  0.2, 6.7  0.2, 5.2  0.20, 7.1  1.0, 13.0  0.2, 2.7  0.5, 16.3  0.4, 8.7  0.5, 6.7  0.2, 4.0  0.5, 10.0  0.1, 4.8  0.5, 7.3 | | | | | | | |
| **Response to diphtheria, tetanus and pneumococcal vaccination** | | | | | | | | | | | | | | | | | | | | | | | | | |
| *Protein vaccine* | | *Criteria* | | | | | | | | | | | *number/total determined (percentage)* | | | | | | | | | | | | |
| Diphtheria | | ≥fourfold increase and above 0.1 (IU/ml)  ≥fourfold increase and above 1.0 (IU/ml) | | | | | | | | | | | 50/77 (65%)  22/77 (29%) | | | | | | | | | | | | |
| Tetanus | | ≥fourfold increase and above 0.1 (IU/ml)  ≥fourfold increase and above 1.0 (IU/ml) | | | | | | | | | | | 58/80 (73%)  55/80 (69%) | | | | | | | | | | | | |
| *Pneumococcal polysaccharide vaccine* | | *Before >0.35 (microg/ml)* | | | | | | | | | | | *After >1.0 (microg/ml)* | | | | | | | | | | | | |
| Serotype 1  Serotype 3  Serotype 4  Serotype 5  Serotype 6  Serotype 7f  Serotype 8  Serotype 9v  Serotype 14  Serotype 15b  Serotype 18c  Serotype 19f  Serotype 20  Serotype 23f  Serotype 33f | | 17/43 (40%)  16/43 (37%)  8/43 (19%)  12/42 (29%)  23/88 (26%)  21/43 (49%)  11/45 (24%)  21/89 (24%)  45/88 (51%)  16/45 (36%)  24/44 (55%)  41/89 (46%)  21/45 (47%)  32/88 (36%)  20/45 (44%) | | | | | | | | | | | 20/44 (55%)  21/43 (49%)  16/43 (37%)  24/43 (56%)  37/85 (44%)  25/44 (57%)  30/42 (71%)  35/86 (41%)  58/86 (67%)  25/43 (58%)  26/43 (60%)  41/86 (48%)  22/43 (51%)  38/86 (44%)  24/43 (56%) | | | | | | | | | | | | |
| The number of serotypes where the after measurements are >1.0 were calculated for each patient; 59 of 86 patients (69%) measured have <7 values >1.0, and are classified as abnormal (laboratory reference values). | | | | | | | | | | | | | | | | | | | | | | | | | |
| **Summaries of categorical laboratory test results** | | | | | | | | | | | | | | | | | | | | | | | | | |
| *Variable* | | | *Category* | | | | | *Number* | | | | | | | | | | *Percentage* | | | | | | | |
| M protein | | | Not present  Present – not monoclonal  Present - monoclonal | | | | | 68  1  0 | | | | | | | | | | 99%  1%  0% | | | | | | | |
| ANA | | | Negative  Positive | | | | | 75  14 | | | | | | | | | | 85%  15% | | | | | | | |
| Rheumatic factor | | | Negative  Positive | | | | | 30  4 | | | | | | | | | | 88%  12% | | | | | | | |
| IgE for tree pollen | | | Class 0  Class 1 - 3  Class 4 - 6 | | | | | 58  6  5 | | | | | | | | | | 84%  9%  7% | | | | | | | |
| IgE for house dust mite | | | Class 0  Class 1 - 3  Class 4 - 6 | | | | | 59  8  2 | | | | | | | | | | 86%  12%  3% | | | | | | | |
| IgE for cat dander | | | Class 0  Class 1 - 3  Class 4 - 6 | | | | | 62  4  3 | | | | | | | | | | 90%  6%  4% | | | | | | | |
| IgE for dog dander | | | Class 0  Class 1 - 3  Class 4 - 6 | | | | | 62  7  0 | | | | | | | | | | 90%  10%  0% | | | | | | | |
| IgE for grass pollen | | | Class 0  Class 1 - 3  Class 4 - 6 | | | | | 57  9  3 | | | | | | | | | | 83%  13%  4% | | | | | | | |
| Aspergillus IgG | | | Negative  Positive | | | | | 16  6 | | | | | | | | | | 73%  27% | | | | | | | |

*The original family history questions were combined to give a more condensed family history. A family history in each of the categories was defined as one or more of the conditions being present. Abbreviations: ANA = antinuclear antibody, BMI = body mass index, COPD = chronic obstructive pulmonary disease, CRP = C-reactive protein, CVID = common variable immunodeficiency disorders, ENT = ear-nose-throat, HRCT = high resolution computed tomography, IBD = inflammatory bowel disease, Ig = immunoglobulin, IVIG = intravenous immunoglobulin substitution, Q = question, SCIG = subcutaneous immunoglobulin substitution, TAAQOL = TNO-AZL Questionnaire for Adult Health-Related Quality of Life [https://www.tno.nl/media/4727/vragenlijsten_01032012.pdf; accessed June 2017].
