# Supplementary material for: Mild Hypogammaglobulinemia Can Be a Serious Condition
Source: Front Immunol. 2018 Oct 15;9:2384. doi: 10.3389/fimmu.2018.02384 (PMC6196282; doi:10.3389/fimmu.2018.02384)
Supplement: Supplementary Table 3 — Statistical analyses in 99 adults with primary antibody deficiency. [file Table_3.docx]

**Supplementary table 3. Statistical analyses in 99 adults with primary antibody deficiency.**

| **Comparisons between referring doctor types** | | | | | | | | | | | | | | | | | | | | | | | | |
| --- | --- | --- | --- | --- | --- | --- | --- | --- | --- | --- | --- | --- | --- | --- | --- | --- | --- | --- | --- | --- | --- | --- | --- | --- |
| *Variable* | *Category* | | | | | *GP* | | | *Internist* | | | | | *Pulmonologist* | | | | | *Other* | | | | | *p-value* |
| Diagnosis | CVID  unPAD | | | | | 3 (9%)  32 (91%) | | | 3 (20%)  12 (80%) | | | | | 3 (7%)  40 (93%) | | | | | 1 (17%)  5 (83%) | | | | | 0.06 |
| HRCT bronchial  wall thickening | No  Yes | | | | | 9 (50%)  9 (50%) | | | 5 (56%)  4 (44%) | | | | | 16 (52%)  15 (48%) | | | | | 1 (50%)  1 (50%) | | | | | 1.00 |
| HRCT bronchiectasis | 0/1 lobes  2+ lobes | | | | | 14 (78%)  4 (22%) | | | 8 (89%)  1 (11%) | | | | | 16 (52%)  15 (48%) | | | | | 2 (100%)  0 (0%) | | | | | 0.08 |
| HRCT mucus plugging | No  Yes | | | | | 16 (89%)  2 (11%) | | | 8 (89%)  1 (11%) | | | | | 24 (77%)  7 (23%) | | | | | 1 (50%)  1 (50%) | | | | | 0.41 |
| HRCT atelectasis | No  Yes | | | | | 15 (83%)  3 (17%) | | | 9 (100%)  0 (0%) | | | | | 24 (77%)  7 (23%) | | | | | 2 (100%)  0 (0%) | | | | | 0.50 |
| **Comparisons between patient diagnoses** | | | | | | | | | | | | | | | | | | | | | | | | |
| *Variable* | | | | *Category* | | | | | | | | | *CVID* | | | | | *unPAD* | | | | | | *p-value* |
| HRCT bronchial wall thickening | | | | No  Yes | | | | | | | | | 3 (43%)  4 (57%) | | | | | 28 (53%)  25 (47%) | | | | | | 0.62 |
| HRCT bronchiectasis | | | | 0/1 lobes  2+ lobes | | | | | | | | | 6 (86%)  1 (14%) | | | | | 34 (64%)  19 (36%) | | | | | | 0.26 |
| HRCT mucus plugging | | | | No  Yes | | | | | | | | | 7 (100%)  0 (0%) | | | | | 42 (79%)  11 (21%) | | | | | | 0.18 |
| HRCT atelectasis | | | | No  Yes | | | | | | | | | 7 (100%)  0 (0%) | | | | | 43 (81%)  10 (19%) | | | | | | 0.21 |
| Therapy (related to antibody deficiency diagnosis) | | | | None  Antibiotic prophylaxis  IVIG or SCIG | | | | | | | | | 0 (0%)  1 (10%)  9 (90%) | | | | | 50 (56%)  19 (21%)  20 (22%) | | | | | | **<0.001** |
| **Differences between patients receiving different therapies** | | | | | | | | | | | | | | | | | | | | | | | | |
| *Variable* | | | | *Category* | | | *None* | | | | | | *Antibiotic prophylaxis* | | | | | *IVIG or SCIG* | | | | | | *p-value* |
| HRCT bronchial wall thickening | | | | No  Yes | | | 10 (42%)  14 (58%) | | | | | | 8 (57%)  6 (43%) | | | | | 13 (59%)  9 (41%) | | | | | | 0.51 |
| HRCT bronchiectasis | | | | 0/1 lobes  2+ lobes | | | 16 (67%)  8 (33%) | | | | | | 7 (50%)  7 (50%) | | | | | 17 (77%)  5 (23%) | | | | | | 0.27 |
| HRCT mucus plugging | | | | No  Yes | | | 20 (83%)  4 (17%) | | | | | | 10 (71%)  4 (29%) | | | | | 19 (86%)  3 (14%) | | | | | | 0.58 |
| HRCT atelectasis | | | | No  Yes | | | 19 (79%)  5 (21%) | | | | | | 11 (79%)  3 (21%) | | | | | 20 (91%)  2 (9%) | | | | | | 0.50 |
| **Comparison of genders** | | | | | | | | | | | | | | | | | | | | | | | | |
| *Variable* | | | *Category* | | | | | | | | | | *Males* | | | | *Females* | | | | | *p-value* | | |
| Diagnosis | | | CVID  UnPAD | | | | | | | | | | 5 (18%)  23 (82%) | | | | 5 (7%)  66 (93%) | | | | | 0.17 | | |
| HRCT bronchial wall thickening | | | No  Yes | | | | | | | | | | 9 (43%)  8 (47%) | | | | 22 (51%)  21 (49%) | | | | | 1.00 | | |
| HRCT bronchiectasis | | | 0/1 lobes  2+ lobes | | | | | | | | | | 10 (59%)  7 (41%) | | | | 30 (70%)  13 (30%) | | | | | 0.55 | | |
| HRCT mucus plugging | | | No  Yes | | | | | | | | | | 13 (76%)  4 (24%) | | | | 36 (84%)  7 (16%) | | | | | 0.71 | | |
| HRCT atelectasis | | | No  Yes | | | | | | | | | | 13 (76%)  4 (24%) | | | | 37 (86%)  6 (14%) | | | | | 0.45 | | |
| Therapy (related to antibody deficiency diagnosis) | | | None  Antibiotic prophylaxis  IVIG or SCIG | | | | | | | | | | 11 (39%)  7 (25%)  10 (36%) | | | | 39 (55%)  13 (19%)  19 (27%) | | | | | 0.37 | | |
| **Differences between most important complaint categories** | | | | | | | | | | | | | | | | | | | | | | | | |
| *Variable* | | *Category* | | | | | | | | *ENT/airway/cough* | | | | | | *Tired/other* | | | | | | | *p-value* | |
| Always ill | | No  Yes | | | | | | | | 4 (8%)  44 (92%) | | | | | | 7 (14%)  43 (86%) | | | | | | | 0.53 | |
| HRCT bronchial wall thickening | | No  Yes | | | | | | | | 16 (50%)  16 (50%) | | | | | | 14 (52%)  3 (48%) | | | | | | | 1.00 | |
| HRCT bronchiectasis | | 0/1 lobes  2+ lobes | | | | | | | | 20 (62%)  12 (38%) | | | | | | 19 (70%)  8 (30%) | | | | | | | 0.59 | |
| HRCT mucus plugging | | No  Yes | | | | | | | | 23 (72%)  9 (28%) | | | | | | 25 (93%)  2 (7%) | | | | | | | **0.05** | |
| HRCT atelectasis | | No  Yes | | | | | | | | 26 (81%)  6 (19%) | | | | | | 23 (85%)  4 (15%) | | | | | | | 0.74 | |
| Gender | | Male  Female | | | | | | | | 12 (25%)  36 (75%) | | | | | | 16 (32%)  34 (68%) | | | | | | | 0.51 | |
| **Associations with being always ill** | | | | | | | | | | | | | | | | | | | | | | | | |
| *Variable* | | *Category* | | | | | | | | *Not always ill* | | | | | | *Always ill* | | | | | | | *p-value* | |
| HRCT bronchial wall thickening | | No  Yes | | | | | | | | 1 (20%)  4 (80%) | | | | | | 29 (54%)  25 (46%) | | | | | | | 0.20 | |
| HRCT bronchiectasis | | 0/1 lobes  2+ lobes | | | | | | | | 4 (80%)  1 (20%) | | | | | | 35 (65%)  19 (35%) | | | | | | | 0.65 | |
| HRCT mucus plugging | | No  Yes | | | | | | | | 5 (100%)  0 (0%) | | | | | | 43 (80%)  11 (20%) | | | | | | | 0.57 | |
| HRCT atelectasis | | No  Yes | | | | | | | | 5 (100%)  0 (0%) | | | | | | 44 (81%)  10 (19%) | | | | | | | 0.58 | |
| Gender | | Male  Female | | | | | | | | 4 (36%)  7 (64%) | | | | | | 24 (28%)  63 (72%) | | | | | | | 0.51 | |
| **Associations with presence of ≥1 of red/swollen joints and/or pain in bones/joints** | | | | | | | | | | | | | | | | | | | | | | | | |
| *Variable* | | *Category* | | | | | | | | *Absence* | | | | | | *Presence* | | | | | | | *p-value* | |
| Always ill | | No  Yes | | | | | | | | 4 (15%)  23 (85%) | | | | | | 7 (10%)  64 (90%) | | | | | | | 0.49 | |
| HRCT bronchial wall thickening | | No  Yes | | | | | | | | 8 (53%)  7 (47%) | | | | | | 22 (50%)  22 (50%) | | | | | | | 1.00 | |
| HRCT bronchiectasis | | 0/1 lobes  2+ lobes | | | | | | | | 9 (60%)  6 (40%) | | | | | | 30 (68%)  14 (32%) | | | | | | | 0.75 | |
| HRCT mucus plugging | | No  Yes | | | | | | | | 10 (67%)  5 (33%) | | | | | | 38 (86%)  6 (14%) | | | | | | | 0.13 | |
| HRCT atelectasis | | No  Yes | | | | | | | | 11 (87%)  4 (13%) | | | | | | 38 (86%)  6 (14%) | | | | | | | 0.25 | |
| Most important complaint | | Ear/airway/cough  Tired/other | | | | | | | | 17 (63%)  10 (37%) | | | | | | 31 (44%)  40 (56%) | | | | | | | 0.11 | |
| Gender | | Male  Female | | | | | | | | 11 (41%)  16 (59%) | | | | | | 17 (24%)  54 (76%) | | | | | | | 0.13 | |
| **Associations with ≥1 of coughing productive, surgery chronic sinusitis, sinusitis nowadays, otitis nowadays, sinusitis in the past, otitis in the past** | | | | | | | | | | | | | | | | | | | | | | | | |
| *Variable* | | *Category* | | | | | | *Absence* | | | | | | | | *Presence* | | | | | | | *p-value* | |
| Always ill | | No  Yes | | | | | | 5 (36%)  9 (64%) | | | | | | | | 6 (7%)  78 (93%) | | | | | | | **0.008** | |
| HRCT bronchial wall thickening | | No  Yes | | | | | | 4 (40%)  6 (60%) | | | | | | | | 26 (53%)  23 (47%) | | | | | | | 0.51 | |
| HRCT bronchiectasis | | 0/1 lobes  2+ lobes | | | | | | 7 (70%)  3 (30%) | | | | | | | | 32 (65%)  17 (35%) | | | | | | | 1.00 | |
| HRCT mucus plugging | | No  Yes | | | | | | 8 (80%)  2 (20%) | | | | | | | | 40 (82%)  9 (18%) | | | | | | | 1.00 | |
| HRCT atelectasis | | No  Yes | | | | | | 9 (90%)  1 (10%) | | | | | | | | 40 (82%)  9 (18%) | | | | | | | 1.00 | |
| Most important complaint | | Ear/airway/cough  Tired/other | | | | | | 5 (%)  9 (64%) | | | | | | | | 43 (51%)  41 (49%) | | | | | | | 0.39 | |
| Gender | | Male  Female | | | | | | 3 (21%)  11 (79%) | | | | | | | | 25 (30%)  59 (70%) | | | | | | | 0.75 | |
| **Associations with season of the year with most complaints** | | | | | | | | | | | | | | | | | | | | | | | | |
| *Variable* | | *Category* | | | | | | *Spring - summer* | | | | | | | | *Autumn - winter* | | | | | | | *p-value* | |
| Family history of allergy | | No  Yes | | | | | | 8 (27%)  22 (73%) | | | | | | | | 18 (27%)  48 (73%) | | | | | | | 1.00 | |
| HRCT bronchial wall thickening | | No  Yes | | | | | | 7 (44%)  9 (56%) | | | | | | | | 21 (51%)  20 (49%) | | | | | | | 0.77 | |
| HRCT bronchiectasis | | 0/1 lobes  2+ lobes | | | | | | 11 (69%)  5 (31%) | | | | | | | | 28 (68%)  13 (32%) | | | | | | | 1.00 | |
| HRCT mucus plugging | | No  Yes | | | | | | 12 (75%)  4 (25%) | | | | | | | | 34 (83%)  7 (17%) | | | | | | | 0.48 | |
| HRCT atelectasis | | No  Yes | | | | | | 13 (81%)  3 (19%) | | | | | | | | 35 (85%)  6 (15%) | | | | | | | 0.70 | |
| **Comparison of TAAQOL domains between Dutch reference population and Care Path data** | | | | | | | | | | | | | | | | | | | | | | | | |
| *Domain* | | *Reference data* | | | | | | | | | | *Care Path data* | | | | | | | | *p-value* | | | | |
| Gross motor functioning  Fine motoric functioning  Cognitive functioning  Sleep  Pain  Social functioning  Daily activities  Sexuality  Vitality  Positive emotions  Depressive emotions  Aggressive emotions | | 100 [81, 100]  100 [100, 100]  93 [75, 100]  81 [56, 94]  81 [63, 94]  88 [75, 100]  100 [75, 100]  100 [75, 100]  67 [50, 83]  67 [58, 75]  83 [67, 92]  89 [78, 100] | | | | | | | | | | 63 [25, 88]  100 [75, 100]  63 [31, 100]  56 [31, 81]  50 [38, 75]  81 [63, 100]  50 [25, 88]  88 [50, 100]  29 [8, 50]  50 [33, 67]  75 [50, 83]  89 [67, 100] | | | | | | | | **<0.001**  **<0.001**  **<0.001**  **<0.001**  **<0.001**  **0.002**  **<0.001**  **0.002**  **<0.001**  **<0.001**  **<0.001**  0.01 | | | | |
| **Associations with ANA positivity** | | | | | | | | | | | | | | | | | | | | | | | | |
| *Variable* | | | | | *Category* | | | | | | *ANA negative* | | | | *ANA positive* | | | | | | *p-value* | | | |
| Bone / joint pain | | | | | No  Yes | | | | | | 20 (27%)  55 (73%) | | | | 3 (23%)  10 (77%) | | | | | | 1.00 | | | |
| Fine motor skills | | | | | - | | | | | | 100 [75, 100] | | | | 100 [75, 100] | | | | | | 0.97 | | | |
| Family history of thyroid disease | | | | | No  Yes | | | | | | 48 (64%)  27 (36%) | | | | 8 (62%)  5 (39%) | | | | | | 1.00 | | | |
| Age | | | | | - | | | | | | 52.1 ± 15.6 | | | | 48.2 ± 16.4 | | | | | | 0.40 | | | |
| Gender | | | | | Male  Female | | | | | | 21 (28%)  54 (72%) | | | | 4 (29%)  10 (71%) | | | | | | 1.00 | | | |
| **Associations with Rheumatoid factor positivity** | | | | | | | | | | | | | | | | | | | | | | | | |
| *Variable* | | | | | *Category* | | | | | | *RF negative* | | | | *RF positive* | | | | | | *p-value* | | | |
| ANA | | | | | Negative  Positive | | | | | | 26 (87%)  4 (13%) | | | | 3 (75%)  1 (25%) | | | | | | 0.49 | | | |
| Bone / joint pain | | | | | No  Yes | | | | | | 1 (3%)  29 (97%) | | | | 1 (25%)  4 (75%) | | | | | | 0.23 | | | |
| Fine motor skills | | | | | - | | | | | | 88 [50, 100] | | | | 78 [53, 91] | | | | | | 0.56 | | | |
| Family history of thyroid disease | | | | | No  Yes | | | | | | 19 (63%)  11 (37%) | | | | 3 (75%)  1 (25%) | | | | | | 1.00 | | | |
| Age | | | | | - | | | | | | 56.6 ± 14.5 | | | | 67.0 ± 6.9 | | | | | | 0.17 | | | |
| Gender | | | | | Male  Female | | | | | | 6 (25%)  24 (80%) | | | | 1 (25%)  3 (75%) | | | | | | 1.00 | | | |
| **Associations with IgE Class 4-6 (IgE positive)** | | | | | | | | | | | | | | | | | | | | | | | | |
| ANA | | | | | Negative  Positive | | | | | | 51 (85%)  9 (15%) | | | | 4 (57%)  3 (43%) | | | | | | 0.10 | | | |
| Rheumatoid factor | | | | | Negative  Positive | | | | | | 23 (96%)  1 (4%) | | | | 1 (100%)  0 (0%) | | | | | | 1.00 | | | |
| Bone / joint pain | | | | | No  Yes | | | | | | 18 (30%)  42 (70%) | | | | 2 (25%)  6 (75%) | | | | | | 1.00 | | | |
| Fine motor skills | | | | | - | | | | | | 100 [84, 100] | | | | 100 [94, 100] | | | | | | 0.38 | | | |
| Family history of thyroid disease | | | | | No  Yes | | | | | | 38 (63%)  22 (37%) | | | | 6 (75%)  2 (25%) | | | | | | 0.70 | | | |
| Age | | | | | - | | | | | | 50.4 ± 15.3 | | | | 42.4 ± 15.8 | | | | | | 0.17 | | | |
| Gender | | | | | Male  Female | | | | | | 16 (26%)  45 (74%) | | | | 3 (37%)  5 (63%) | | | | | | 0.66 | | | |

Abbreviations: ANA = antinuclear antibody, CVID = common variable immunodeficiency disorders, GP = general practitioner, HRCT = high resolution computed tomography, Ig = immunoglobulin, IVIG = intravenous immunoglobulin, RF = rheumatoid factor, SCIG = subcutaneous immunoglobulin, TAAQOL = TNO-AZL Questionnaire for Adult Health-Related Quality of Life [https://www.tno.nl/media/4727/vragenlijsten_01032012.pdf; accessed June 2017], unPAD = unclassified antibody deficiency according to European Society for Immunodeficiencies (ESID) Registry criteria [https://esid.org/Working-Parties/Registry/Diagnosis-criteria; accessed June 2017]. P-values in bold are considered significant (<0.01).
