# Supplementary material for: Mild Hypogammaglobulinemia Can Be a Serious Condition
Source: Front Immunol. 2018 Oct 15;9:2384. doi: 10.3389/fimmu.2018.02384 (PMC6196282; doi:10.3389/fimmu.2018.02384)
Supplement: Supplementary Table 4 — Clustering analyses in 99 adults with primary antibody deficiency. [file Table_4.docx]

**Supplementary table 4. Clustering analyses in 99 adults with primary antibody deficiency.**

| Analyses using the K-means clustering method with the variables age, female gender, smoking packyears, HRCT - bronchial wall thickening / 2+ lobes bronchiectasis / mucus plugging / atelectasis, TAAQOL domain scores, IgG, IgA, IgM, IgG1, IgG2, IgG3, IgG4, postvaccination titers to diphtheria / tetanus, number of pneumococcal serotypes <1.00 microg/ml postvaccination in the data analysis. Only patients with complete information on all variables could be included, and thus the analysis was based on 51 patients. | | | | |
| --- | --- | --- | --- | --- |
| *Number of clusters* | | *Calinski/Harabasz pseudo-F index* | | |
| **2**  3  4  5 | | **24.8**  15.7  11.2  10.8 | | |
| *Comparisons of variables among the patient groups in the two-cluster solution*  *{Figures are mean ± standard deviation, median [inter-quartile range] or number (percentage)}* | | | | |
| *Variable* | *Cluster 1 (n=30)* | | *Cluster 2 (n=21)* | *p-value* |
| Age  Female gender  Smoking packyears | 53.0 ± 14.7  23 (77%)  6 [0, 20] | | 55.8 ± 15.2  16 (76%)  14 [5, 20 | 0.50  1.00  0.19 |
| Bronchial wall  2+ lobes bronchiectasis  Mucus plugging  Atelectasis | 9 (30%)  15 (50%)  8 (27%)  1 (3%) | | 6 (29%)  13 (62%)  7 (33%)  1 (5%) | 1.00  0.57  0.76  1.00 |
| Gross motor functioning  Fine motoric functioning  Cognitive functioning  Sleep  Pain  Social functioning  Daily activities  Sexuality  Vitality  Positive emotions  Depressive emotions  Aggressive emotions | 75 [50, 100]  100 [88, 100]  93 [75, 100]  81 [69, 100]  63 [38, 88]  94 [75, 100]  75 [63, 100]  100 [88, 100]  42 [25, 67]  67 [50, 75]  79 [67, 92]  100 [89, 100] | | 31 [6, 44]  88 [63, 100]  31 [13, 50]  31 [19, 50]  38 [19, 56]  56 [31, 69]  25 [13, 44]  38 [25, 88]  8 [0, 25]  33 [16, 58]  42 [33, 67]  67 [56, 100] | **<0.001**  0.21  **<0.001**  **<0.001**  **0.005**  **<0.001**  **<0.001**  **<0.001**  **<0.001**  **0.003**  **<0.001**  **0.005** |
| IgG  IgA  IgM  IgG1  IgG2  IgG3  IgG4 | 7.7 [5.9, 9.6]  1.3 [0.7, 2.8]  0.7 [0.3, 1.2]  5.2 [3.9, 6.5]  1.5 [1.1, 2.5]  0.3 [0.2, 0.4]  0.14 [0.07, 0.44] | | 7.0 [5.9, 7.8]  1.4 [0.7, 1.8]  0.5 [0.4, 0.8]  4.9 [4.0, 5.9]  1.2 [0.9, 1.6]  0.3 [0.2, 0.5]  0.15 [0.07, 0.28] | 0.39  0.80  0.25  0.25  0.14  0.99  0.97 |
| Diphtheria (post)  Tetanus (post)  Number pneumococcal serotypes <1 (post) | 0.6 [0.1, 1.3]  6.0 [1.9, 14.1]  4.2 ± 2.9 | | 0.6 [0.4, 1.3]  9.9 [3.9, 16.0]  4.8 ± 2.6 | 0.52  0.33  0.49 |
| CVID diagnosis  unPAD diagnosis | 5 (17%)  25 (83%) | | 1 (5%)  20 (95%) | 0.38 |
| Analyses using the K-means clustering method with the same variables but omitting the HRCT-related variables in the data analysis, which had the most missing data. This analysis was based on 75 patients. | | | | |
| *Number of clusters* | | *Calinski/Harabasz pseudo-F index* | | |
| **2**  3  4  5 | | **36.7**  24.9  20.6  16.9 | | |

| *Comparisons without HRCT-related variables among the patient groups in the two-cluster solution*  *{Figures are mean ± standard deviation, median [inter-quartile range] or number (percentage)}* | | | |
| --- | --- | --- | --- |
| *Variable* | *Cluster 1 (n=38)* | *Cluster 2 (n=37)* | *p-value* |
| Age  Female gender  Smoking packyears | 50.9 ± 15.3  30 (79%)  4 [0, 18] | 50.9 ± 16.1  28 (76%)  8 [0, 19] | 0.99  0.79  0.29 |
| Gross motor functioning  Fine motoric functioning  Cognitive functioning  Sleep  Pain  Social functioning  Daily activities  Sexuality  Vitality  Positive emotions  Depressive emotions  Aggressive emotions | 84 [63, 100]  100 [88, 100]  97 [75, 100]  75 [63, 100]  63 [50, 88]  100 [88, 100]  81 [63, 100]  100 [88, 100]  46 [33, 75]  67 [50, 75]  83 [67, 92]  100 [89, 100] | 44 [13, 63]  88 [75, 100]  31 [19, 50]  44 [25, 56]  38 [19, 56]  63 [44, 75]  25 [6, 38]  50 [25, 75]  8 [0, 25]  33 [25, 50]  58 [33, 67]  77 [97, 89] | **<0.001**  0.08  **<0.001**  **<0.001**  **<0.001**  **<0.001**  **<0.001**  **<0.001**  **<0.001**  **<0.001**  **<0.001**  **<0.001** |
| IgG  IgA  IgM  IgG1  IgG2  IgG3  IgG4 | 7.4 [6.0, 9.5]  1.2 [0.6, 2.2]  0.8 [0.3, 1.4]  5.0 [3.9, 6.6]  1.4 [1.1, 2.2]  0.3 [0.2, 0.4]  0.13 [0.08, 0.25] | 7.5 [6.2, 9.6]  1.6 [1.1, 2.4]  0.5 [0.4, 0.8]  5.1 [4.4, 6.5]  1.5 [1.2, 2.2]  0.4 [0.2, 0.5]  0.27 [0.07, 0.42] | 0.56  0.22  0.16  0.16  0.66  0.28  0.14 |
| Diphtheria (post)  Tetanus (post)  Number pneumococcal serotypes <1 (post) | 0.7 [0.2, 1.5]  6.8 [2.2, 16.0]  4.6 ± 3.0 | 0.6 [0.3, 0.9]  9.9 [3.6, 16.0]  5.5 ± 2.6 | 0.89  0.26  0.14 |
| CVID diagnosis  unPAD diagnosis | 5 (13%)  33 (87%) | 1 (3%)  36 (97%) | 0.20 |

Abbreviations: CVID = common variable immunodeficiency disorders, HRCT = high resolution computed tomography, Ig = immunoglobulin, TAAQOL = TNO-AZL Questionnaire for Adult Health-Related Quality of Life [https://www.tno.nl/media/4727/vragenlijsten_01032012.pdf; accessed June 2017], unPAD = unclassified antibody deficiency according to European Society for Immunodeficiencies (ESID) Registry criteria [https://esid.org/Working-Parties/Registry/Diagnosis-criteria; accessed June 2017]. P-values in bold are considered significant (<0.01).
